# The Chemically Highly Diversified Metabolites from the Red Sea Marine Sponge *Spongia* sp.

**DOI:** 10.3390/md20040241

**Published:** 2022-03-30

**Authors:** Chi-Jen Tai, Atallah F. Ahmed, Chih-Hua Chao, Chia-Hung Yen, Tsong-Long Hwang, Fang-Rong Chang, Yusheng M. Huang, Jyh-Horng Sheu

**Affiliations:** 1 Doctoral Degree Program in Marine Biotechnology, National Sun Yat-sen University, Kaohsiung 80424, Taiwan; chijentai@g-mail.nsysu.edu.tw; 2 Department of Pharmacognosy, College of Pharmacy, King Saud University, Riyadh 11451, Saudi Arabia; afahmed@ksu.edu.sa; 3 Department of Pharmacognosy, Faculty of Pharmacy, Mansoura University, Mansoura 35516, Egypt; 4 School of Pharmacy, China Medical University, Taichung 406040, Taiwan; chchao@mail.cmu.edu.tw; 5 Chinese Medicine Research and Development Center, China Medical University Hospital, Taichung 406040, Taiwan; 6 Graduate Institute of Natural Products, College of Pharmacy, Kaohsiung Medical University, Kaohsiung 80708, Taiwan; chyen@kmu.edu.tw (C.-H.Y.); aaronfrc@kmu.edu.tw (F.-R.C.); 7 Graduate Institute of Natural Products, College of Medicine, Chang Gung University, Taoyuan 333, Taiwan; htl@mail.cgu.edu.tw; 8 Research Center for Chinese Herbal Medicine, Graduate Institute of Healthy Industry Technology, College of Human Ecology, Chang Gung University of Science and Technology, Taoyuan 333, Taiwan; 9 Department of Anesthesiology, Chang Gung Memorial Hospital, Taoyuan 333, Taiwan; 10 Department of Marine Recreation, National Penghu University of Science and Technology, Magong 880011, Taiwan; yusheng@gms.npu.edu.tw; 11 Tropical Island Sustainable Development Research Center, National Penghu University of Science and Technology, Magong 880011, Taiwan; 12 Department of Marine Biotechnology and Resources, National Sun Yat-sen University, Kaohsiung 804, Taiwan; 13 Department of Medical Research, China Medical University Hospital, China Medical University, Taichung 404333, Taiwan

**Keywords:** Red Sea sponge, *Spongia* sp., halogenated labdane diterpenoid, polyoxygenated steroid, fatty acid, polychlorinated metabolites, cytotoxicity, antibacterial assay, anti-inflammatory assay

## Abstract

A polyoxygenated and halogenated labdane, spongianol (**1**); a polyoxygenated steroid, 3β,5α,9α-trihydroxy-24*S*-ethylcholest-7-en-6-one (**2**); a rare seven-membered lactone B ring, (22*E*,24*S*)-ergosta-7,22-dien-3β,5α-diol-6,5-olide (**3**); and an α,β-unsaturated fatty acid, (*Z*)-3-methyl-9-oxodec-2-enoic acid (**4**) as well as five known compounds, 10-hydroxykahukuene B (**5**), pacifenol (**6**), dysidamide (**7**), 7,7,7-trichloro-3-hydroxy-2,2,6-trimethyl-4-(4,4,4-trichloro-3-methyl-1-oxobu-tylamino)-heptanoic acid methyl ester (**8**), and the primary metabolite 2’-deoxynucleoside thymidine (**9**), have been isolated from the Red Sea sponge *Spongia* sp. The stereoisomer of **3** was discovered in *Ganoderma resinaceum*, and metabolites **5** and **6**, isolated previously from red algae, were characterized unprecedentedly in the sponge. Compounds **7** and **8** have not been found before in the genus *Spongia*. Compounds **1**–**9** were also assayed for cytotoxicity as well as antibacterial and anti-inflammatory activities.

## 1. Introduction

**Figure 1:**
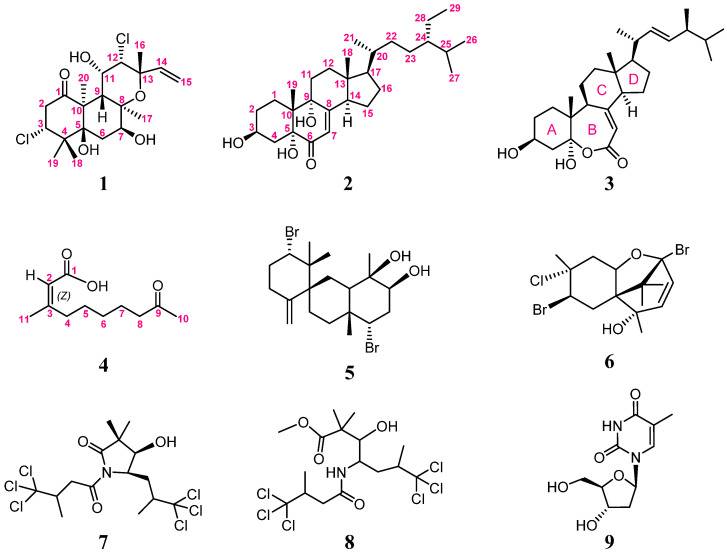
Structures of metabolites **1**–**9**.

Sponges of the genus *Spongia* are known to be abundant sources of chemical constituents with diverse structures and bioactivity [1,2]. So far, the 3,4-*seco*-3,19-dinorditerpenes [3], 5,5,6,6,5-pentacyclic diterpenes [4], furanoterpenes [5,6], spongian diterpenes [7,8], scalarane sesterterpenoids [9,10], sesquiterpene quinones [11,12], diverse terpenes [13,14], sterols [15,16], and macrolides [14,17], along with fatty acids [18,19] and halides [20,21], have been isolated from the genus *Spongia* sp. Our preliminary studies on the Red Sea sponge *Spongia* sp. resulted in the isolation of a series of new compounds, including one 5,5,6,6,5-pentacyclic diterpene, two new furanotrinorsesquiterpenoid acids, and a furanyl trinorsesterpenoid [22]. Our continuous investigation of the chemical constituents of this sponge has again afforded four new compounds, including spongianol (**1**), 3β,5α,9α-trihydroxy-24*S*-ethylcholest-7-en-6-one (**2**), (22*E*,24*S*)-ergosta-7,22-dien-3β,5α-diol-6,5-olide (**3**), and (*Z*)-3-methyl-9-oxodec-2-enoic acid (**4**) (Figure 1), along with five known metabolites: 10-hydroxykahukuene B (**5**) [23], pacifenol (**6**) [24], dysidamide (**7**) [25], 7,7,7-trichloro-3-hydroxy-2,2,6-trimethyl-4-(4,4,4-trichloro-3-methyl-1-oxobutylamino)-heptanoic acid methyl ester (**8**) [25], and the primary metabolite 2’-deoxynucleoside thymidine (**9**) [26]. The molecular structures of **1**–**9** were established by MS, IR, and detailed NMR spectroscopic analysis (Supplementary Figures S1–S45) and by comparison with the reported spectral data of related known compounds. The cytotoxicity of hepatocellular carcinoma (HCC) Huh7 cells and the anti-inflammatory and antibacterial activity of **1**–**9** were also evaluated.

## 2. Results and Discussion

**Figure 2:**
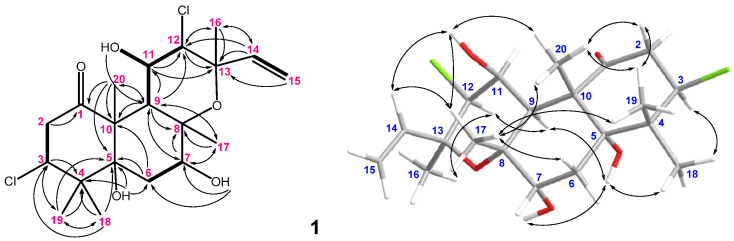
The selected COSY (▬), HMBC (→), and key NOESY (↔) correlations of **1**.

Compound **1** was obtained as a colorless oil. The HRESIMS (Supplementary Figure S1) of **1** established the molecular formula C_20_H_3__0_^35^Cl_2_O_5_, implying five degrees of unsaturation. The IR spectrum of **1** revealed the presence of the hydroxyl, carbonyl, and olefin from absorptions at 3420, 1698, and 1646 cm^–1^, respectively. The ^13^C NMR spectroscopic data of **1** exhibited 20 carbon signals (Table 1), which were designated with the assistance of the DEPT spectrum as five methyls (δ_C_ 28.7, 25.9, 24.0, 20.1, and 18.9), three methylenes (including δ_C_ 116.7, 45.1, and 27.7), six methines (including four oxymethines, δ_C_ 70.9, 75.3, 68.9, and 65.9; one terminal vinyl group methine, δ_C_ 139.9; and δ_C_ 41.7), and five quaternary carbons (including one ketone carbon, δ_C_ 209.2; three oxygenated quaternary carbons, δ_C_ 82.1, 77.4, and 76.2; and δ_C_ 45.2). The NMR data of **1** (Table 1) showed the appearance of a vinyl group (*δ*_C_ 139.9, CH and 116.7, CH_2_; *δ*_H_ 6.78, 1H, dd, *J* = 17.5 and 11.5 Hz; and *δ*_H_ 5.35, 1H, d, *J* = 17.5 Hz, and 5.21, 1H, d, *J* = 11.5 Hz, respectively). The ^1^H–^1^H COSY experiment revealed the presence of four partial structures (Figure 2). The HMBC correlations of **1** (Figure 2) displayed from H_3_-20 (δ_H_ 1.71) to C-1, 5, 9, and 10 (δ_C_ 209.2, 82.1, 41.7, and 58.1); both H_3_-18 and 19 (δ_H_ 1.24 and 1.21) to C-3, 4 (δ_C_ 65.9 and 45.2), and 5; H_3_-17 (δ_H_ 1.66) to C-7, 8 (δ_C_ 75.3 and 76.2), and 9; H_3_-16 (δ_H_ 1.49) to C-12, 13, and 14 (δ_C_ 68.9, 77.4, and 139.9) suggested that the five methyls were positioned at C-4, 8, 10, and 13. Furthermore, the HMBC correlations were observed from both H_3_-20 and H_2_-2 (δ_H_ 3.34 and 2.64, each 1H) to a ketone carbon (δ_C_ 209.2); H-3 (δ_H_ 4.50) to C-2 (δ_C_ 45.1), 4, 18 (δ_C_ 24.0), and 19 (δ_C_ 20.1); 5-OH (δ_H_ 5.72) to C-4, 5 and 6; H-7 (δ_H_ 3.67) to C-6 (δ_C_ 27.7), 8, 9, and 17 (δ_C_ 25.9); 7-OH (δ_H_ 3.59) to C-6 and 7; H-9 (δ_H_ 2.72) to C-7, 8, 10, 11, 12 and 20; H-11 (δ_H_ 5.32) to C-12 and 13; 11-OH (δ_H_ 2.25) to C-9 and 11; H-12 (δ_H_ 4.06) to C-13 and 16 (δ_C_ 28.7); both H-14 (δ_H_ 6.78) and H_2_-15 (δ_H_ 5.35 and 5.21, each 1H) to C-13, suggesting that a ketone, three hydroxyls, and one terminal vinyl functionalities were located on C-1, 5, 7, 11, and 14, respectively. The remaining two chlorines were positioned at C-3 and 12 (δ_C_ 65.9 and 68.9, respectively). As described above, **1** elucidated a new polyoxygenated chlorinated labdane diterpenoid, spongianol.

**Figure 3:**
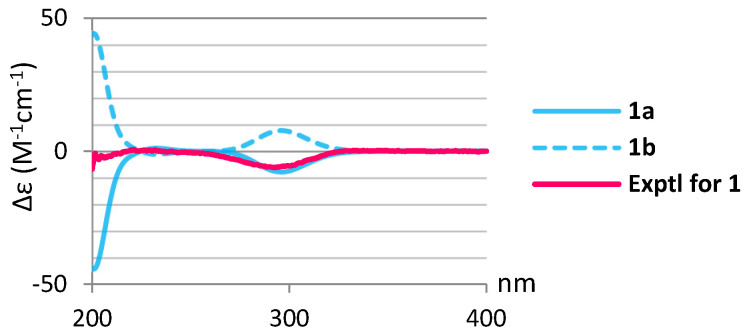
Calculated ECD curves of 3*R*,5*R*,7*S*,8*R*,9*R*,10*S*,11*S*,12*S*,13*S*-**1** (**1a**) and 3*S*,5*S*,7*R*,8*S*,9*S*,10*R*,11*R*,12*R*,13*R*-**1** (**1b**) and the experimental CD curve of **1**.

**Figure 4:**
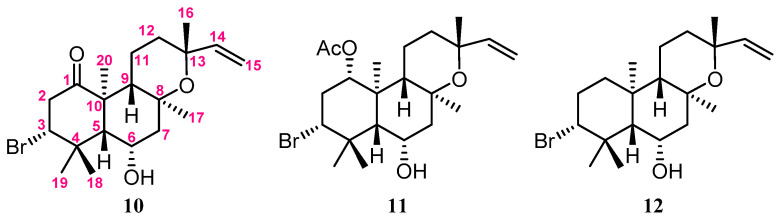
The structural analogs of **10**–**12** [29,30].

In the NOESY spectrum of **1**, the NOE correlations (Figure 2) of H_3_-20 with 11-OH, H_3_-17, and H_3_-19; 11-OH and H_3_-19 with H_3_-17; and H-14 with 11-OH and H_3_-17 suggested that these protons were positioned at the same orientation. By contrast, the correlations of H-3 with H_3_-18; 5-OH with H-9, H_3_-18, and 7-OH; and H-12 with H-9 and H_3_-16 revealed that these protons were on the same side. Furthermore, the stereochemistry of **1** was evidenced by the experimental CD (circular dichroism) and calculated ECD (electronic circular dichroism) spectra (Figure 3). The theoretical ECD curves of 3*R*,5*R*,7*S*,8*R*,9*R*,10*S*,11*S*,12*S*,13*S*-**1** (**1a**) and its enantiomer 3*S*,5*S*,7*R*,8*S*,9*S*,10*R*,11*R*,12*R*,13*R*-**1** (**1b**) were calculated at the B3LYP/6-311+G(d,p) (including a IEFPCM solvent model for MeOH) level of theory by the Gaussian 9.0 program [27,28]. The CD spectrum of **1** (Figure 3) showed the negative Cotton effect at 293 nm, which was found to be consistent with the calculated ECD of **1a** (296 nm, Figure 3), and the absolute configuration of **1** was thus identified as 3*R*,5*R*,7*S*,8*R*,9*R*,10*S*,11*S*,12*S* and 13*S*. Furthermore, the absolute configurations of **1** were consistent with that of the structural analogs (3*R*,5*S*,6*S*,8*S*,9*S*,10*R*,13*R*)-3-bromo-6-hydroxy-8,13-epoxylabd-14-en-1-one (**10**), (1*S*,3*R*,5*S*, 6*S*,8*S*,9*S*,10*R*,13*R*)-1-acetoxy-3-bromo-6-hydroxy-8,13-epoxy-labd-14-ene (**11**) and paniculatol (**12**) (Figure 4) isolated from the red alga *Laurencia* sp. [29,30].

**Figure 5:**
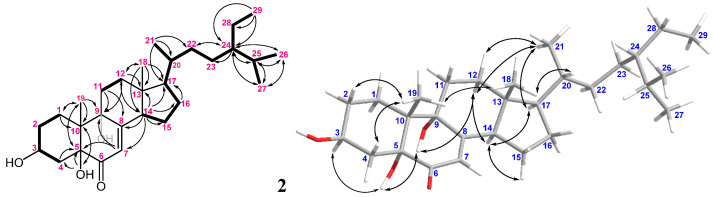
The selected COSY (▬), HMBC (→), and key NOESY (↔) correlations of **2**.

**Figure 6:**
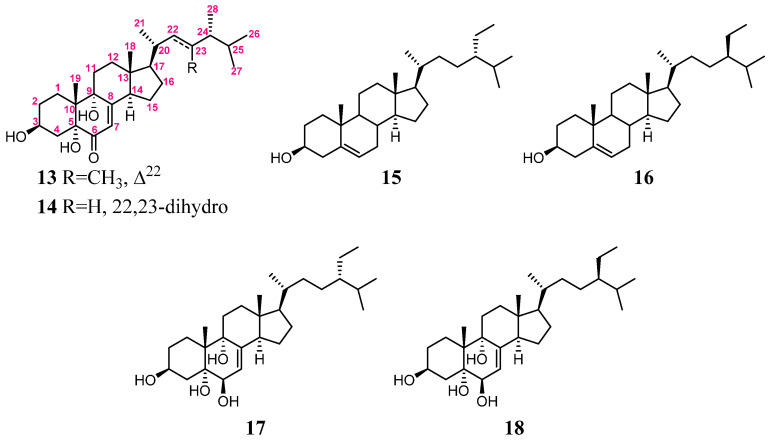
Structures of compounds **13**–**18**.

The HRESIMS of metabolite **2** showed a molecular ion peak [M + Na]^+^ at 483.3446 *m*/*z*, which established the molecular formula C_2__9_H_4__8_O_4_, implying six degrees of unsaturation. The IR absorption bands at ν_max_ 3458–3291, 1682, and 1654 cm^−^^1^ revealed the presence of the hydroxyl, ketone, and olefin, respectively. The NMR spectroscopic data of **2** (Table 1) displayed six methyls (δ_C_ 20.5, 19.6, 19.0, 18.9, 12.3, and 12.0; δ_H_ 1.02, 3H, s; 0.84, 3H, d, *J* = 6.6 Hz; 0.81, 3H, d, *J* = 6.6 Hz; 0.94, 3H, d, *J* = 6.0 Hz; 0.85, 3H, t, *J* = 7.8 Hz and 0.61, 3H, s); six methylenes; five methines (including one oxygenated methane δ_C_ 67.2 and δ_H_ 4.07, m; and one olefinic methane δ_C_ 119.9 and δ_H_ 5.66, br s), and five quaternary carbons (including one ketone carbon δ_C_ 197.7; one olefinic non-protonated carbon δ_C_ 164.3, and two oxygenated quaternary carbons δ_C_ 79.7 and 74.6, respectively). The detailed analyses of ^1^H–^1^H COSY and HMBC correlations (Figure 5) established the molecular skeleton of **2**. Furthermore, a comparison of the NMR data of **2** with the similar structures 3β,5α,9α-trihydroxy-(22*E*,24*R*)-23-methylergosta-7,22-dien-6-one (**13**) and 3β,5α,9α-trihydroxy-(24*S*)-ergost-7-en-6-one (**14**) [31] (Figure 6, Supplementary Table S1) confirmed that **2** is a new polyoxygenated sterol.

**Figure 7:**
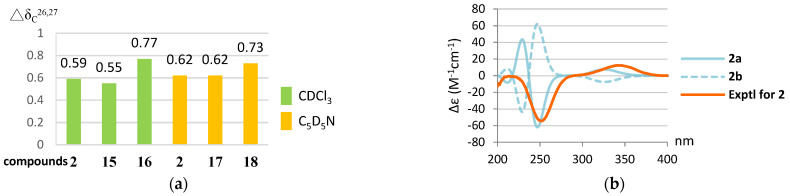
(**a**) The Δδ_C_^26,27^ values of compounds **2** and **15**–**18**; (**b**) the calculated ECD curves of 3*S*,5*R*,9*R*,10*R*,13*R*,14*R*,17*R*,20*R*,24*S*-**2** (**2a**) and 3*R*,5*S*,9*S*,10*S*,13*S*,14*S*,17*S*,20*S*,24*R*-**2** (**2b**), and the experimental CD curve of **2**.

The relative configuration of **2** was deduced by the analysis of NOE correlations (Figure 5). The observation of the NOE correlations of 5-OH (δ_H_ 3.34) with H-3 and 9-OH (δ_H_ 4.07 and 4.12, respectively); 9-OH with H-12α (δ_H_ 1.72); H-14 (δ_H_ 2.72) with H-12α and H-17 (δ_H_ 1.39); H-17 with H_3_-21 (δ_H_ 0.94); and H_3_-21 with H_2_-12 elucidated that these protons were cofacial. Furthermore, a comparison of the NMR data of **2** with those of similar analogs, **13** and **14** (Figure 6), confirmed the configuration of the steroidal nucleus ([31], Supplementary Table S1). The chemical shift differences of C-26 and C-27 (Δδ_C_^26,27^, useful for assignment of the absolute configuration of C-24 of this side chain [32,33,34]) for compound **2** and the related known 24*S* and 24*R* analogs are summarized in Supplementary Table S2. In 24*S* analogues **15** (CDCl_3_) and **17** (C_5_D_5_N), the Δδ_C_^26,27^ values were found to be 0.55 and 0.62, respectively, while those for 24*R* analogues **16** (CDCl_3_) and **18** (C_5_D_5_N) were 0.77 and 0.73, respectively (Figure 6) [32,35]. The Δδ_C_^26,27^ values for **2** were found to be 0.59 and 0.62 in CDCl_3_ and C_5_D_5_N, respectively, indicating that C-24 of **2** had an *S*-configuration (Figure 7a). In addition, the δ_C_ values for C-20, C-24, to C-27 were shown to be much more similar to those of compound **15** than those of **16**. Thus, the 24*S* configuration of **2** was established. Furthermore, the stereochemistry of **2** was evidenced by the experimental CD and calculated ECD spectra (Figure 7b). The theoretical ECD curves of 3*S*,5*R*,9*R*,10*R*,13*R*,14*R*,17*R*,20*R*,24*S*-**2** (**2a**) and its enantiomer 3*R*,5*S*,9*S*,10*S*,13*S*,14*S*,17*S*,20*S*,24*R*-**2** (**2b**) were calculated at the CAM-B3LYP/6-311+G(d,p) (including a IEFPCM solvent model for MeOH) level of theory by the Gaussian 9.0 program [27,28]. The tendency of the experimental CD spectrum of **2** (Figure 7b) was similar to that of the calculated ECD of **2a** (Figure 7b); furthermore, the absolute configuration of **2** was identified. On the basis of the above observations, the molecular structure of **2** was determined as 3β,5α,9α-trihydroxy-24*S*-ethylcholest-7-en-6-one.

**Figure 8:**
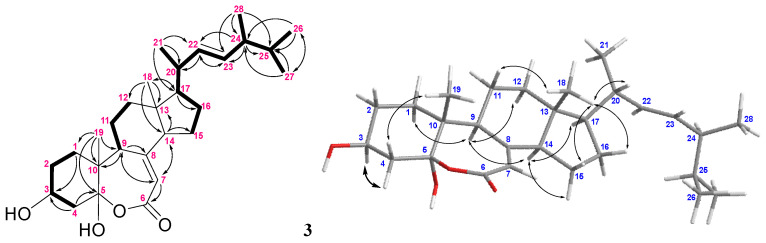
The selected COSY (▬), HMBC (→), and key NOESY (↔) correlations of **3**.

**Figure 9:**
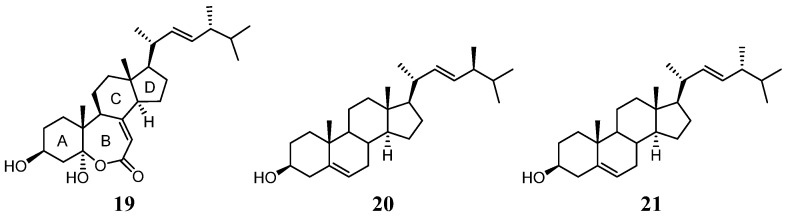
Structures of compounds **19**–**21**.

For compound **3**, the IR absorption bands at ν_max_ 3393 and 1670 cm^−^^1^ revealed the presence of the hydroxy and α,β-unsaturated ester groups, respectively, and the HRESIMS *m*/*z* 445.3316 [M + H]^+^ established the molecular formula of **3** to be C_2__8_H_44_O_4_. The structure of **3** was established by analyses of ^1^H–^1^H COSY and HMBC experiments (Figure 8), and the planar structure of **3** was found to be as same as that of (22*E*,24*R*)-ergosta-7, 22-dien-3β,5α-diol-6,5-olide (**19**) (Figure 9) [36]. Compound **3** exhibited almost the same NMR data as those of **19** except for the chemical shifts of C-16 and C-26 to C-28 (Supplementary Table S3). The chemical shifts of C-16 and C-24 to C-28 for **3** and the related known 24*S* and 24*R* analogs **19**–**21** [32,36] are summarized in Table 2. It was found that C-16 of **3** resonated at a higher chemical shift (δ_C_ 28.0) than that of **19** (δ_C_ 27.7). The 0.3 ppm difference for carbon shifts of C-16 between both **3** and **19** was similar to those of **20** (δ_C_ 28.86) and **21** (δ_C_ 28.58). In addition, the chemical shifts of C-24 and C-26−28 of **3** showed closer chemical shift values to compound **20** than to **21**. On the basis of the above analysis, compound **3** was determined as (22*E*,24*S*)-ergosta-7,22- dien-3β,5α-diol-6,5-olide. Compound **3** is the fourth member of the group of steroids with a rare seven-membered lactone B ring, and the other three members, astersterol A, fortisterol, and (22*E*,24*R*)-ergosta-7,22-dien-3β,5α-diol-6,5-olide, were isolated previously from the starfish [37], the marine sponge [38], and the fungus *Ganoderma resinaceum* [36], respectively.

**Figure 10:**
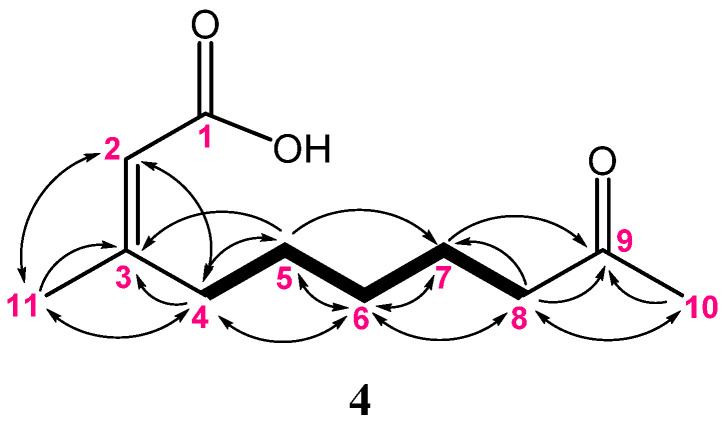
The selected COSY (▬) and HMBC (→) correlations of **4**.

Metabolite **4** was isolated as a colorless oil. Its molecular formula was determined to be C_11_H_18_O_3_ from the HRESIMS (*m/z* 221.1149 [M + Na]^+^), indicating three degrees of unsaturation. The IR spectrum displayed the absorptions of the hydroxyl, ketone, and carboxylic acid groups (3445, 1700, and 1683 cm^–1^, respectively). The NMR data (Table 3) showed the presence of two methyls (δ_C_ 29.9 and 25.4; δ_H_ 2.14 and 1.91, each 3H, s); five methylenes; one alkene methane (δ_C_ 115.3 and δ_H_ 5.69, br s); and three quaternary carbons (included one ketone carbon δ_C_ 209.4, one carbonyl carbon δ_C_ 169.5, and one alkene quaternary carbon δ_C_ 163.5). The detailed analysis of ^1^H–^1^H COSY and HMBC correlations of **4** (Figure 10) assigned the positions of a carboxylic acid group, an olefinic double bond, and ketone functionalities to be at C-1, C-2, and C-9, respectively. Moreover, the NOE correlation observed for H-2 (δ_H_ 5.69) with H_3_-11 (δ_H_ 1.91) in **4** suggested the *Z* geometry of this double bond and consequently established the structure of **4** to be (*Z*)-3-methyl- 9-oxodec-2-enoic acid. 

10-Hydroxykahukuene B (**5**) [23] is a brominated diterpene with a rare prenylated chamigrane skeleton. To the best of our knowledge, two examples of this skeleton have been reported in the marine red alga *Laurencia* sp. [23,39,40], and **5** represent the first example of a metabolite with a prenylated chamigrane skeleton that has been isolated from the sponge. Pacifenol (**6**), the first trihalogenated compound with a chamigrane skeleton, was isolated by Sims and associates from the Californian red alga *Laurencia pacifica* [41]. After that, pacifenol was also isolated from other marine red algae, including *L. caduciramulosa* [42] and *L*. *marianensis* [24], among others. Mollusks of the genus *Aplysia* are known to be animals that do not biosynthesize the halogenated sesquiterpenes by themselves but obtain and accumulate these metabolites by ingesting alga and, in some cases, transform the alga metabolites into other compounds in the digestive gland [43,44]. Our present study is the first report to discover pacifenol in the sponge. Metabolites **7** and **8** have also been isolated from the Red Sea sponges *Dysidea herbacea* [45] and *Lamellodysidea herbacea* [25], but they were discovered for the first time in sponges of the genus *Spongia* in the present study.

It was previously known that sponges could take in and accumulate organohalides from environmental seawater and that these compounds might be transformed into chemical defense substances [46]. Macroalgae are important primary producers in coral reefs, and many species inhabit areas near sponges [47,48]. Algae synthesize secondary metabolites for competition and survival [49,50,51], and the red alga *Laurencia* sp. is known for producing diverse halides, many of which have been shown to have antibacterial activity [52,53]. The sponges could inhale these halides or even transform them chemically into compounds such as **1**, **5**, and **6** for their own use [46].

Compounds **1**–**9** were tested for cytotoxicity using a resazurin assay in the HCC Huh7 cell line. Among them, compounds **5** and **8** showed weak cytotoxicity against the Huh7 cell line, with 17% and 32% inhibition toward the proliferation of Huh7 cells at 50 μM, respectively. Furthermore, **5** and **8** could inhibit the 43% and 53% proliferation of Huh7 cells at 200 μM, respectively. The growth inhibition assay of *Staphylococcus aureus* (*S. aureus*) was subsequently applied for compounds **1**–**9**. The results showed that **9** displayed 31%, 37%, and 89% inhibition on the growth of *S. aureus* at 50, 100, and 200 µM, respectively. 

The anti-inflammatory activities of compounds **1**–**9** inhibiting superoxide anion (O_2_^−^) generation and elastase release in fMLF/CB-stimulated human neutrophils [54,55,56] were also evaluated (Table 4). Compounds **7** and **8** exhibited medium inhibitory activity against elastase release (55.96 ± 3.88 and 60.80 ± 6.49%, respectively) at 20 μM, with IC_50_ values of 17.23 ± 2.45 and 14.60 ± 2.24 μM, respectively. However, compounds **7** and **8** showed weak inhibition of superoxide anion generation (25.24 ± 4.68% and 22.38 ± 3.95%, respectively) at 20 μM. Furthermore, compounds **6** and **9** were found to display inhibitory activity to some extent (20.00 ± 4.87% and 21.22 ± 4.71%, respectively) against elastase release at 20 μM.

## 3. Materials and Methods

### 3.1. General Experimental Procedures

Measurements of circular dichroisms, optical rotations, and IR spectra were carried out on a Jasco J-715 CD spectrometer, JASCO P-1020 polarimeter, and FT/IR-4100 infrared spectrophotometer (JASCO Corporation, Tokyo, Japan), respectively. ESIMS was performed on a Bruker APEX II (Bruker, Bremen, Germany) mass spectrometer, and HRESIMS was performed on a Bruker APEX II and Impact HD Q-TOF mass spectrometers (Bruker, Bremen, Germany). The NMR spectra were recorded on a Varian 400MR FT-NMR at 400 and 100 MHz for ^1^H and ^13^C, respectively; a Varian Unity INOVA500 FT-NMR (both Varian Inc., Palo Alto, CA, USA) at 500 and 125 MHz for ^1^H and ^13^C, respectively; or a JEOL ECZ600R FT-NMR (Japan) at 600 and 150 MHz for ^1^H and ^13^C, respectively. Silica gel and reversed-phase (RP-18, 230–400 mesh) silica gel were used for column chromatography and analytical thin-layer chromatography (TLC) analysis (Kieselgel 60 F-254, 0.2 mm, Merck, Darmstadt, Germany), respectively. The isolation and purification of compounds by high-performance liquid chromatography (HPLC) were achieved using a Hitachi L-2455 HPLC apparatus (Hitachi, Tokyo, Japan) equipped with a Supelco C18 column (250 × 21.2 mm, 5 μm, Supelco, Bellefonte, PA, USA).

### 3.2. Animal Material

The sponge *Spongia* sp. was collected in March 2016 off the Red Sea coast of Jeddah, Saudi Arabia (21°22′11.08″ N, 39°06′56.62″ E). A voucher sample (RSS-1) was deposited at the Department of Pharmacognosy, College of Pharmacy, King Saud University, Saudi Arabia.

### 3.3. Extraction and Separation

The freeze-dried material *Spongia* sp. (550 g dry wt) was minced and extracted exhaustively with EtOAc/MeOH/CH_2_Cl_2_ (1:1:0.5). The solvent-free extract was suspended in water and partitioned with CH_2_Cl_2_, EtOAc, and then *n*-BuOH saturated with water to obtain CH_2_Cl_2_ (18.47 g), EtOAc (0.78 g), and *n*-BuOH (1.0 g) fractions. The CH_2_Cl_2_ fraction was chromatographed over a silica gel column using EtOAc in *n*-hexane (0% to 100%, stepwise) and then MeOH in EtOAc (0% to 100%, stepwise) to yield 12 fractions (F1–F12).

Fraction F3 (0.986 g) eluted with *n*-hexane/EtOAc (9:1) was re-chromatographed over a RP-18 column using H_2_O in MeOH (100% to 0%, stepwise) to give six subfractions (F3-1 to F3-6). F3-5 (54.6 mg, eluted with MeOH/H_2_O 8:2) was isolated using RP-18 HPLC (MeOH/H_2_O 9:1) to give six subfractions (F3-5-1 to F3-5-6); F3-5-2 (32.5 mg) was further purified on RP-18 HPLC (MeOH/H_2_O 7.5:2.5) to afford **6** (1.6 mg). 

F5 (1.706 g) eluted with *n*-hexane/EtOAc (6.5:3.5) was re-chromatographed over a RP-18 column using H_2_O in MeOH (100% to 0%, stepwise) to give eight subfractions (F5-1 to F5-8). F5-4 (32.4 mg, eluted with MeOH/H_2_O 6:4) was isolated using RP-18 HPLC (MeOH/H_2_O 7:3) to give eight subfractions (F5-4-1 to F5-4-8). F5-4-1 (29.5 mg) was further purified on RP-18 HPLC (CH_3_CN /H_2_O 4:6) to afford **7** (5.8 mg) and **8** (1.7 mg). F5-6 (48.2 mg, eluted with MeOH/H_2_O 1:0) was separated on RP-18 HPLC (IPA/MeOH 1:19) to give six subfractions (F5-6-1 to F5-6-6), F5-6-1 (27.6 mg) was purified on RP-18 HPLC (MeOH/H_2_O 8:2) to afford **1** (2.9 mg) and **5** (4.6 mg).

F7 (1.505 g) eluted with *n*-hexane/EtOAc (2.5:7.5) was re-chromatographed over a RP-18 column using H_2_O in MeOH (100% to 0%, stepwise) to give eight subfractions (F7-1 to F7-8). F7-3 (146.3 mg, eluted with MeOH/H_2_O 4:6) was isolated using RP-18 HPLC (MeOH/H_2_O 1:1) to give 10 subfractions (F7-3-1 to F7-3-10). F7-3-7 (13.2 mg) was purified on RP-18 HPLC (CH_3_CN/H_2_O 2.8:7.2) to afford **4** (4.5 mg). F7-6 (67.0 mg, eluted with MeOH/H_2_O 1:0) was isolated using RP-18 HPLC (isopropanol/MeOH 1:19) to give nine subfractions (F7-6-1 to F7-6-9); F7-6-5 (16.1 mg) was further separated on RP-18 HPLC (MeOH/H_2_O 93:7) to afford **3** (1.0 mg); F7-6-7 (25.7 mg) was purified on RP-18 HPLC (CH_3_CN/H_2_O 8:2) to afford **2** (4.9 mg).

#### 3.3.1. Spongianol (**1**)

Colorless oil; ${\left[\alpha \right]}_{25}^{D}$ − 14.5 (*c* 0.29, CH_3_OH); IR (neat) *v*_max_ 3420, 2979, 2919, 1698, and 1646 cm^−1^; ^1^H and ^13^C NMR data, see Table 1; ESIMS *m*/*z* 443 and 445 [M + Na]^+^; HRESIMS *m*/*z* 443.1364 and [M + Na]^+^ (calcd for C_20_H_3__0_^35^Cl_2_O_5_Na, 443.1363).

#### 3.3.2. 3β,5α,9α-Trihydroxy-24*S*-ethylcholest-7-en-6-one (**2**)

White powder; ${\left[\alpha \right]}_{25}^{D}$ − 12.7 (*c* 0.49, CH_3_OH); IR (neat) *v*_max_ 3291, 2959, 2872, 1682 and 1654 cm^−1^; ^1^H and ^13^C NMR data, see Table 1; ESIMS *m*/*z* 483 [M + Na]^+^; HRESIMS *m*/*z* 483.3446 [M + Na]^+^ (calcd for C_2__9_H_48_O_4_Na, 483.3445).

#### 3.3.3. (22*E*,24*S*)-Ergosta-7,22-dien-3β,5α-diol-6,5-olide (**3**)

Colorless crystal; ${\left[\alpha \right]}_{25}^{D}$ + 38.5 (*c* 0.28, CH_3_OH); IR (neat) ν_max_ 3393, 2954, 2917, 2849, and 1670 cm^–1^; ^1^H NMR and ^13^C data, see Table 1; HRESIMS *m/z* 445.3316 [M + H]^+^ (calcd for C_28_H_45_O_4_, 445.3312).

#### 3.3.4. (*Z*)-3-Methyl-9-oxodec-2-enoic Acid (**4**)

Colorless oil; IR (neat) ν_max_ 3445, 2923, 2859, 1700, 1683, and 1647 cm^−1^; ^1^H and ^13^C NMR data, see Table 3; HRESIMS *m/z* 221.1149 [M + Na]^+^ (calcd for C_11_H_18_O_3_Na, 221.1148).

### 3.4. DFT and TD-DFT Calculations

The preliminary geometry optimization of conformers was simulated using the DFT approach at the B3LYP/6-31G(d) level of theory [27]. The ECD spectra were simulated by using the time-dependent DFT (TD-DFT) approach at the B3LYP/6-311+G(d,p) or CAM-B3LYP/6-311+G(d,p) level of theory. The range-separated functional CAM-B3LYP is recommended for ECD calculations [27]. The bulk solvent effect of methanol was taken into account with the integral equation formalism polarizable continuum model (IEFPCM solvent model for MeOH). All calculations were performed by the Gaussian 09 program [28]. The calculated ECD curves were converted using GaussSum 2.2.5 and illustrated using Microsoft Excel.

### 3.5. Cytotoxicity Assay 

The cytotoxicity assay was performed using the methods described in a previous paper [57,58]. Huh7 cells were cultured in a 96-well plate containing 100 μL of culture medium in triplicate and treated with indicated concentrations of compounds for 72 h. At the assay time point, resazurin (Cayman Chemical) was added and incubated for 4 h at 37 °C. The DMSO wells was defined as the control and assigned a relative cell viability of 100%. Sorafenib, the positive control, inhibited the 52% proliferation of Huh7 cells at 12.5 µM.

### 3.6. Antibacterial Assay

The antibacterial assay was performed using the methods described in a previous paper [59]. *S. aureus* was cultured in Lysogeny broth (LB) in a shaker–incubator at 37 °C for 24 h. The cultures were then diluted to an absorbance at 600 nm of 0.04 using sterile LB. The diluted bacteria aliquots were placed (100 μL per well) into 96-well flat-bottom plates. Tested compounds (cpd) were then added to the final concentration at 50 μM, 100 μM, and 200 μM, respectively. Background controls (1% DMSO in LB solution), positive controls (1% DMSO in the diluted bacteria solution), and known drug controls (tetracyclin; inhibited the 99% growth of bacteria at 50 µM) were run on the same plate. The absorbance at 600 nm (A) was measured right after the testing compounds were added for the basal absorbance and after 16 h incubation at 37 °C. The percentage bacterial growth was calculated as follows: [(Acpd − Acpd_basal) − Abackground control]/[(Apositive control − Apositive control_basal) − Abackground control] × 100. 

### 3.7. Anti-inflammatory Activity

Human neutrophils were isolated from the blood of healthy adult volunteers and enriched by using dextran sedimentation, Ficoll–Hypaque gradient centrifugation, and hypotonic lysis, as described previously [56]. Then, neutrophils were incubated in Ca^2+^-free HBSS buffer (pH 7.4, ice-cold). 

#### 3.7.1. Superoxide Anion Generation

Neutrophils (6 × 10^5^ cells/mL) incubated (with 0.6 mg/mL ferricytochrome *c* and 1 mM Ca^2+^) in HBSS at 37 °C were treated with DMSO (as a control) or the tested compound for 5 min. Neutrophils were primed with 1 μg/mL cytochalasin B (CB) for 3 min before being activated by 100 nM fMLF for 10 min. The change in superoxide anion generation was spectrophotometrically measured at 550 nm (U-3010, Hitachi, Tokyo, Japan) [54,55]. LY294002 [2-(4-morpholinyl)-8-phenyl-1(4*H*)-benzopyran-4-one] was used as a positive control.

#### 3.7.2. Elastase Release

Neutrophils (6 × 10^5^ cells/mL) incubated (with 100 μM MeO-Suc-Ala-Ala-Pro-Val-*p*-nitroanilide and 1 mM Ca^2+^) in HBSS at 37 °C were treated with DMSO or the tested compound for 5 min. Neutrophils were, then, activated with fMLF (100 nM)/CB (0.5 μg/mL) for 10 min. The change in elastase release was spectrophotometrically measured at 405 nm (U-3010, Hitachi, Tokyo, Japan) [55].

## 4. Conclusions

New metabolites (**1**–**4**) along with five known compounds (**5**–**9**) were isolated from a Red Sea sponge, *Spongia* sp. Compounds **5** and **8** showed weak cytotoxicity to HCC Huh7 cells, while **9** displayed significant inhibition against *S. aureus*. Furthermore, compounds **7** and **8** exhibited notable activity to inhibit elastase release and weaker inhibitory activity toward superoxide anion generation. Both compounds **6** and **9** also showed inhibition against elastase release. Although compound **5** was found to be inactive in the present study, its antibacterial activity against *S. aureus* and *E. coli* has been reported [23]. It is noteworthy that some of the isolates from this sponge were also found in red algae, which suggests that the specific metabolites of sponges could have originated from alga and would be accumulated and/or transformed into metabolites in sponges.

## Figures and Tables

**Table 1:** ^13^C and ^1^H NMR data for compounds **1**–**3**.

1 ^a^			2 ^b^			3 ^b^		
#	δ_H_	δ_C_	#	δ_H_	δ_C_	#	δ_H_	δ_C_
1	–	209.2, C ^c^	1	1.52, m	25.1, CH_2_	1	1.95, m	35.9, CH_2_
2α	2.64, dd (14.0, 6.0) ^d^	45.1, CH_2_		2.34, dt (11.0, 3.0)			1.40, m	
						2α	1.86, m	30.4, CH_2_
2β	3.34, t (13.0)		2α	1.93, m	30.1, CH_2_	2β	1.40, m	
3	4.50, dd (13.0, 6.0)	65.9, CH	2β	1.51, m		3	3.79, m	67.6, CH
			3	4.07, m	67.2, CH	4α	2.25, m	46.2. CH_2_
4	–	45.2, C	4α	2.09, dd (11.5, 1.5)	37.2, CH_2_	4β	2.10, m	
						5	–	104.3, C
5	–	82.1, C	4β	1.77, m		6	–	166.4, C
6α	2.18, tt (15.5, 3.5)	27.7, CH_2_	5	–	79.7, C	7	5.70, br s	115.3, CH
			6	–	197.7, C	8	–	159.7, C
6β	2.05, tt (15.5, 3.5)		7	5.66, br s	119.9, CH	9	2.26, m	51.7, CH
			8	–	164.3, C	10	–	43.1, C
7	3.67, t (3.5)	75.3, CH	9	–	74.6, C	11	1.82, m	25.3, CH_2_
8	–	76.2, C	10	–	41.8, CH		1.64, m	
9	2.72, br s ^e^	41.7, CH	11	1.76, m	28.8, CH_2_	12α	2.05, m	39.9, CH_2_
10	–	58.1, C		1.94, m		12β	1.42, m	
11	5.32, t (2.5)	70.9, CH	12α	1.72, m	35.0, CH	13	–	46.6, C
12	4.06, d (2.5)	68.9, CH	12β	1.92, m		14	2.14, m	58.1, CH
13	–	77.4, C	13	–	45.4, C	15α	1.58, m	23.1, CH_2_
14	6.78, dd (17.5, 11.5)	139.9, CH	14	2.72, dd (9.5, 4.5)	51.7, C	15β	1.50, m	
						16	1.77, m	28.0, CH_2_
15	5.21, d (11.5)	116.7, CH_2_	15α	1.64, m	22.5, CH_2_		1.33, m	
	5.35, d (17.5)		15β	1.51, m		17	1.37, m	56.3, CH
16	1.49, s	28.7, CH_3_	16	1.40, m	27.7, CH_2_	18	0.62, s	12.4, CH_3_
17	1.66, s	25.9, CH_3_		2.01, m		19	1.06, s	17.8, CH_3_
18	1.24, s	24.0, CH_3_	17	1.39, m	56.0, CH	20	2.05, m	40.4, CH
19	1.21, s	20.1, CH_3_	18	0.61, s	12.0, CH_3_	21	1.01, d (6.0)	21.0, CH_3_
20	1.71, s	18.9, CH_3_	19	1.02, s	20.5, CH_3_	22	5.13, dd (15.0, 7.8)	135.2, CH
5-OH	5.72, br s		20	1.40, m	36.4, CH	23	5.21, dd (15.0, 8.4)	132.7, CH
7-OH	3.59, d (2.0)		21	0.94, d (6.0)	18.9, CH_3_	24	1.85, m	43.1, CH
11-OH	2.25, d (1.5)		22	1.05, m	33.7, CH_2_	25	1.47, m	33.2, CH
				1.39, m		26	0.82, d (6.6)	19.6, CH_3_
			23	1.38, m	26.3, CH_2_	27	0.84, d (7.2)	20.1, CH_3_
			24	0.93, m	46.0, CH	28	0.92, d (7.2)	18.0, CH_3_
			25	1.63, m	28.9, CH			
			26	0.81, d (6.6)	19.0, CH_3_			
			27	0.84, d (6.6)	19.6, CH_3_			
			28	1.14, m	23.0, CH_2_			
				1.32, m				
			29	0.85, t (7.8)	12.3, CH_3_			
			5-OH	3.34 br s	–			
			9-OH	4.12 br s	–			

^a^^13^C and ^1^H spectra recorded at 125 and 500 MHz in CDCl_3_; ^b 13^C and ^1^H spectra recorded at 150 and 600 MHz in CDCl_3_; ^c^ deduced from DEPT; ^d^ *J* values (Hz) in parentheses; ^e^ broad signal.

**Table 2:** The ^13^C NMR data at C-16 and C-24–C-28 of **3** and related compounds **19**−**21**.

Position	3 ^a^ (22*E*,24*S*)	19 ^b^ (22*E*,24*R*)	20 ^c^ (22*E*,24*S*)	21 ^c^ (22*E*,24*R*)
C-16	28.0	27.7	28.86	28.58
C-24	43.1	42.8	43.12	42.90
C-25	33.2	33.0	33.28	33.16
C-26	19.6	19.9	19.69	20.02
C-27	20.1	19.6	20.19	19.69
C-28	18.0	17.6	18.08	17.68

^a 13^C spectra recorded at 150 MHz in CDCl_3_; ^b 13^C spectra recorded at 150 MHz in CDCl_3_ [36]; ^c 13^C spectra recorded at 25.16 MHz in CDCl_3_ [32].

**Table 3:** ^13^C and ^1^H NMR data for compound **4**.

4		
Position	δ_H_	δ_C_
1	-	169.5, C ^a^
2	5.69, br s ^b^	115.3, CH
3	-	163.5, C
4	2.62, t (7.5) ^c^	33.1, CH_2_
5	1.48, quin (7.5)	27.8, CH_2_
6	1.33, quin (7.5)	29.0, CH_2_
7	1.60, quin (7.5)	23.5, CH_2_
8	2.43, t (7.5)	43.6, CH_2_
9	-	209.4, C
10	2.14, s	29.9, CH_3_
11	1.91, s	25.4, CH_3_

^13^C and ^1^H spectra recorded at 125 and 500 MHz in CDCl_3_. ^a^ Deduced from DEPT; ^b^ broad signal; ^c^ *J* values (Hz) in parentheses.

**Table 4:** Effects of compounds on superoxide anion generation and elastase release in fMLF/CB-induced human neutrophils.

Compound	Superoxide Anion		Elastase Release	
	IC_50_ (μM) ^a^	Inh% (20 μM)	IC_50_ (μM)	Inh% (20 μM)
**1**	>20	15.65 ± 7.56	>20	16.31 ± 4.66 *
**2**	>20	−0.04 ± 3.90	>20	6.28 ± 3.04
**3**	>20	18.10 ± 2.29 **	>20	13.08 ± 2.01 **
**4**	>20	7.81 ± 3.87	>20	18.53 ± 3.57 **
**5**	>20	3.25 ± 4.06	>20	13.27 ± 3.81 *
**6**	>20	15.51 ± 7.55	>20	20.00 ± 4.87 *
**7**	>20	25.24 ± 4.68 **	17.23 ± 2.45	55.96 ± 3.88 ***
**8**	>20	22.38 ± 3.95 **	14.60 ± 2.24	60.80 ± 6.49 ***
**9**	>20	15.58 ± 0.58 ***	>20	21.22 ± 4.71 *
LY294002	1.91 ± 0.79	88.71 ± 1.50 ***	2.94 ± 0.13	79.50 ± 1.95 ***

Results are presented as mean ± S.E.M. (*n* = 3). * *p* < 0.05, ** *p* < 0.01, *** *p* < 0.001 compared with the control value (DMSO). ^a^ Concentration necessary for 50% inhibition.

## Data Availability

Data of the present study are available in the article and supplementary materials.

## Supplementary Materials

The following supporting information can be downloaded at: https://www.mdpi.com/article/10.3390/md20040241/s1, Table S1: Selected ^1^H and ^13^C NMR data of **2** and similar compounds **13** and **14** in CDCl_3_ and C_5_D_5_N, Table S2: Selected ^13^C NMR data at C20–C29 of **2** and related compounds **15**–**18**, Table S3: ^13^C and ^1^H NMR data of **3** and related compound **19**, Table S4: Cytotoxicity of compounds **1**–**9**, Table S5: The cartesian coordinates of conformer of compound **1** at the B3LYP/6-311+G(d,p) level of theory, Table S6: The cartesian coordinates of conformer of compound **2** at the CAM-B3LYP/6-311+G(d,p) level of theory, Figures S1–S30: 1D and 2D NMR spectra and HRESIMS spectra of compounds **1**–**4**, Figures S31–S45: ^1^H and ^13^C NMR spectra and LR- or HR-ESIMS of compounds **5**–**9**.
