# Supplementary material for: The Chemically Highly Diversified Metabolites from the Red Sea Marine Sponge Spongia sp
Source: Mar Drugs. 2022 Mar 30;20(4):241. doi: 10.3390/md20040241 (PMC9028682; doi:10.3390/md20040241)
Supplement: Supplementary file 1 [file marinedrugs-20-00241-s001.zip › marinedrugs-1629372-supplementary.pdf]

The Chemically Highly Diversified Metabolites from a Red Sea Marine Sponge *Spongia* sp.

Chi-Jen Tai,[1,†] Atallah F. Ahmed,[2,3,†] Chih-Hua Chao,[4,5] Chia-Hung Yen,[6] Tsong-Long Hwang,[7,8,9] Fang-Rong Chang,[6] Yusheng M. Huang[10,11] and Jyh-Horng Sheu[1,6,12,13,*]

1 Doctoral Degree Program in Marine Biotechnology, National Sun Yat-sen University, Kaohsiung 80424, Taiwan; chijentai@g-mail.nsysu.edu.tw (C.-J.T.).
2 Department of Pharmacognosy, College of Pharmacy, King Saud University, Riyadh 11451, Saudi Arabia; afahmed@ksu.edu.sa (A.F.A.).
3 Department of Pharmacognosy, Faculty of Pharmacy, Mansoura University, Mansoura 35516, Egypt
4 School of Pharmacy, China Medical University, Taichung 40604, Taiwan; chchao@mail.cmu.edu.tw (C.-H.C.)
5 Chinese Medicine Research and Development Center, China Medical University Hospital, Taichung 40604, Taiwan
6 Graduate Institute of Natural Products, College of Pharmacy, Kaohsiung Medical University, Kaohsiung 80708, Taiwan; chyen@kmu.edu.tw; (C.-H.Y.), aaronfrc@kmu.edu.tw (F.-R.C.)
7 Graduate Institute of Natural Products, College of Medicine, Chang Gung University, Taoyuan 333, Taiwan; htl@mail.cgu.edu.tw (T.-L.H.)
8 Research Center for Chinese Herbal Medicine, Graduate Institute of Healthy Industry Technology, College of Human Ecology, Chang Gung University of Science and Technology, Taoyuan 333, Taiwan
9 Department of Anesthesiology, Chang Gung Memorial Hospital, Taoyuan 333, Taiwan
10 Department of Marine Recreation, National Penghu University of Science and Technology, Magong, Penghu 88046, Taiwan; yusheng@gms.npu.edu.tw (Y.M.H.).
11 Tropical Island Sustainable Development Research Center, National Penghu University of Science and Technology, Magong, Penghu 88046, Taiwan.
12 Department of Marine Biotechnology and Resources, National Sun Yat-sen University, Kaohsiung 80424, Taiwan
13 Department of Medical Research, China Medical University Hospital, China Medical University, Taichung 404333, Taiwan.

* Correspondence: sheu@mail.nsysu.edu.tw (J.-H.S.); Tel.: +886-7-525-2000 (ext. 5030); Fax: +886-7-525-5020.
† These authors contributed equally to this work.

Contents

Table S1. Selected ^{1}H and ^{13}C NMR data of **2** and similar compounds **13** and **14** in $CDCl_3$ and C_5D_5N. 3
Table S2. Selected ^{13}C NMR data at C20-C29 of **2** and related compounds **15**−**18**........ 3
Table S3. ^{13}C and ^{1}H NMR data of **3** and related compound **19**........ 4
Table S4. Cytotoxicities of compounds **1**−**9**. 5
Table S5. The cartesian coordinates of conformer of compound **1** at the B3LYP/6-311+G(d,p) level of theory........11
Table S6. The cartesian coordinates of conformer of compound **2** at the CAM-B3LYP/6-311+G(d,p) level of theory........ 16

Figure S1. HRESIMS spectrum of **1**. 6
Figure S2. ^{1}H NMR spectrum of **1**. 6
Figure S3. ^{13}C NMR spectrum of **1**. 7
Figure S4. DEPT spectrum of **1**........ 7
Figure S5. HSQC spectrum of **1** 8
Figure S6. ^{1}H−^{1}H COSY spectrum of **1**........ 8
Figure S7. HMBC spectrum of **1**........ 9
Figure S8. The partial HMBC spectrum of **1**........ 9
Figure S9. NOESY spectrum of **1**. 10
Figure S10. HRESIMS spectrum of **2**. 12
Figure S11. ^{1}H NMR spectrum of **2**. 12
Figure S12. ^{13}C NMR spectrum of **2**. 13
Figure S13. HSQC spectrum of **2**........ 13
Figure S14. ^{1}H−^{1}H COSY spectrum of **2**........ 14
Figure S15. HMBC spectrum of **2**........ 14
Figure S16. NOESY spectrum of **2**. 15
Figure S17. HRESIMS spectrum of **3**. 18
Figure S18. ^{1}H NMR spectrum of **3**. 18
Figure S19. ^{13}C NMR spectrum of **3**. 19
Figure S20. ^{1}H−^{1}H COSY spectrum of **3**. 19
Figure S21. HMBC spectrum of **3**........ 20
Figure S22. NOESY spectrum of **3**. 20
Figure S23. HRESIMS spectrum of **4**. 21
Figure S24. ^{1}H NMR spectrum of **4**. 21
Figure S25. ^{13}C NMR spectrum of **4**. 22
Figure S26. DEPT spectrum of **4**........ 22
Figure S27. HSQC spectrum of **4**........ 23
Figure S28. ^{1}H−^{1}H COSY spectrum of **4**........ 23
Figure S29. HMBC spectrum of **4**........ 24
Figure S30. NOESY spectrum of **4**. 24
Figure S31. HRESIMS spectrum of **5**. 25
Figure S32. ^{1}H NMR spectrum of **5**. 25
Figure S33. ^{13}C NMR spectrum of **5**. 26
Figure S34. ESIMS spectrum of **6**........ 26
Figure S35. ^{1}H NMR spectrum of **6**. 27
Figure S36. ^{13}C NMR spectrum of **6**. 27

Figure S37. HRESIMS spectrum of **7**. 28
Figure S38. ^{1}H NMR spectrum of **7**. 28
Figure S39. ^{13}C NMR spectrum of **7**. 29
Figure S40. HRESIMS spectrum of **8**. 29
Figure S41. ^{1}H NMR spectrum of **8**. 30
Figure S42. ^{13}C NMR spectrum of **8**. 30
Figure S43. ESIMS spectrum of **9**. 31
Figure S44. ^{1}H NMR spectrum of **9**. 31
Figure S45. ^{13}C NMR spectrum of **9**. 32

Table S1. Selected ^{1}H and ^{13}C NMR data of **2** and similar compounds **13** and **14** in $CDCl_3$ and C_5D_5N.

position	**2**			**13**			**14**		
	δ_H ^a^	δ_H ^b^	δ_C ^b^	δ_H ^c^	δ_H ^d^	δ_C ^d^	δ_H ^c^	δ_H ^d^	δ_C ^d^
3	4.07 m	4.68 m	67.2 CH	4.02 m	4.65 m	66.8 CH	4.06 m	4.65 m	66.8 CH
5	–	–	80.2 C	–	–	79.8 C	–	–	79.8 C
6	–	–	199.6 C	–	–	199.2 C	–	–	199.2 C
7	5.66 br s [e]	5.96 d (1.5)	120.7 CH	5.67 d (2.1)	5.95 d (1.8)	120.3 CH	5.67 d (2.0)	5.93 d (1.8)	120.3 CH
8	–		164.6 C	–	–	164.2 C	–	–	164.2 C
9	–		75.4 C	–	–	75.1 C	–	–	75.0 C
10	–		42.7 C	–	–	42.3 C	–	–	42.3 C
14	2.72 m	3.00 m	52.3 CH	2.74 m	3.00 m	52.0 CH	2.74 m	2.97 m	51.9 CH
17	1.39 m	1.37 m	56.7 CH			57.0 CH			56.3 CH
21	0.94 d (6.0)	0.99 d (4.5)	19.5 CH_3	0.96 d (6.6)	1.00 d (6.6)	20.3 CH_3	0.94 d (5.8)	1.00 d (6.6)	19.2 CH_3
OH-3	–	–	–	–	6.34 d (4.8)	–	–	6.33 d (4.8)	–
OH-5	3.34 br s	8.66 br s	–	–	8.63 s	–	–	8.62 s	–
OH-9	4.12 br s	6.31 br s	–	–	6.32 br s	–	–		–

a. ^{1}H spectra recorded at 600 MHz in $CDCl_3$; b. ^{13}C and ^{1}H spectra recorded at 125 and 500 MHz in C_5D_5N; c. ^{1}H spectra recorded at 270 MHz in $CDCl_3$ [31]; d. ^{13}C and ^{1}H spectra recorded at 150 and 600 MHz in C_5D_5N [31]; e. Broad signal.

Table S2. Selected ^{13}C NMR data at C20-C29 of **2** and related compounds **15**−**18**.

position	**2**	**15** (24*S*)	**16** (24*R*)	**2**	**17** (24*S*)	**18** (24*R*)
	δ_C ^a^	δ_C ^b^	δ_C ^b^	δ_C ^c^	δ_C ^d^	δ_C ^d^
20-CH	36.44	36.29	36.17	37.23	37.05	36.91
21-CH_3	18.86	18.82	18.82	19.53	19.16	19.16
22-CH_2	33.68	33.95	33.95	34.42	34.11	34.16
23-CH_2	26.35	26.43	26.13	27.16	26.78	26.50
24-CH	46.00	46.07	45.85	46.74	46.32	46.09
25-CH	28.90	28.98	29.18	29.72	29.28	29.51
26-CH_3	18.96	19.07	19.84	19.61	19.16	20.00
27-CH_3	19.55	19.62	19.07	20.23	19.78	19.27
28-CH_2	23.03	23.09	23.09	23.83	23.34	23.39
29-CH_3	12.29	12.32	12.32	12.96	12.52	12.17

a. ^{13}C spectra recorded at 150 MHz in $CDCl_3$; b. ^{13}C spectra recorded at 25.16 MHz in $CDCl_3$ [32]. c. ^{13}C spectra recorded at 150 MHz in C_5D_5N; d. ^{13}C spectra recorded at 149.9 MHz in C_5D_5N [35].

Table S3. ^{13}C and ^{1}H NMR data of **3** and related compound **19**.

	(22*E*,24*S*)-Ergosta-7,22-dien-3β,5α-diol-6,5-olide (**3**) [a]		(22*E*,24*R*)-Ergosta-7,22-dien-3β,5α-diol-6,5-olide (**19**) [b]	
position	δ_H	δ_C	δ_H	δ_C
1	1.95, m	35.9, CH_2	1.93, m	35.8, CH_2
	1.40, m		1.40, m	
2	1.86, m	30.4, CH_2	1.85, m	30.4, CH_2
	1.40, m		1.40, m	
3	3.79, m	67.6, CH	3.79,m	67.6, CH
4	2.25, m	46.2. CH_2	2.28, m,	46.1, CH_2
	2.10, m		2.11, m	
5	–	104.3, C	–	104.3, C
6	–	166.4, C	–	166.6, C
7	5.70, br s [c]	115.3, CH	5.69, s	115.2, CH
8	–	159.7, C	–	159.4, C
9	2.26, m	51.7, CH	2.25, m	51.7, CH
10	–	43.1, C	–	42.8, C
11	1.82, m	25.3, CH_2	1.82, m	25.3, CH_2
	1.64, m		1.64, m	
12	2.05, m	39.9, CH_2	2.05, m	39.9, CH_2
	1.42, m		1.41, m	
13	–	46.6, C	–	46.6, C
14	2.14, m	58.1, CH	2.13, m	58.0, CH
15	1.58, m	23.1, CH_2	1.53, m	23.0, CH_2
	1.50, m		1.49, m	
16	1.77, m	28.0, CH_2	1.76, m	27.7, CH_2
	1.33, m		1.36, m	
17	1.37, m	56.3, CH	1.35, m	56.4, CH
18	0.62, s	12.4, CH_3	0.62, s	12.5, CH_3
19	1.06, s	17.8, CH_3	1.05, s	17.7, CH_3
20	2.05, m	40.4, CH	2.03, m	40.3, CH
21	1.01, d (6.0) [d]	21.0, CH_3	1.01, d (6.6)	21.0, CH_3
22	5.13, dd (15.0, 7.8)	135.2, CH	5.14, dd (15.2, 8.5)	135.0, CH
23	5.21, dd (15.0, 8.4)	132.7, CH	5.23, dd (15.3, 7.8)	132.6, CH
24	1.85, m	43.1, CH	1.85, m	42.8, CH
25	1.47, m	33.2, CH	1.47, m	33.0, CH
26	0.82, d (6.6)	19.6, CH_3	0.84, d (6.9)	19.9, CH_3
27	0.84, d (7.2)	20.1, CH_3	0.82, d (6.8)	19.6, CH_3
28	0.92, d (7.2)	18.0, CH_3	0.91, d (6.8)	17.6, CH_3
OH-5			3.99, brs	

[a.] ^{13}C and ^{1}H spectra recorded at 150 and 600 MHz in $CDCl_3$; [b.] ^{13}C and ^{1}H spectra recorded at 150 and 600 MHz in $CDCl_3$ [36]; [c.] Broad signal; [d.] *J* values (Hz) in parentheses.

Table S4. Cytotoxicity of compounds **1**−**9**.

Compound	Huh7 cell viability		
	12.5 μM (%, mean±SD)	50 μM (%, mean±SD)	200 μM (%, mean±SD)
**1**	–[a]	–	–
**2**	–	–	–
**3**	–	–	–
**4**	–	–	–
**5**	–	83.0±3.8	56.8±2.3
**6**	–	–	–
**7**	–	–	–
**8**	–	68.1±1.1	46.6±0.7
**9**	–	100.2±2.7	97.7±2.3
Sorafenib	47.7±0.3		

[a] viability %>100. Results are presented as mean ± SD. (n = 3).

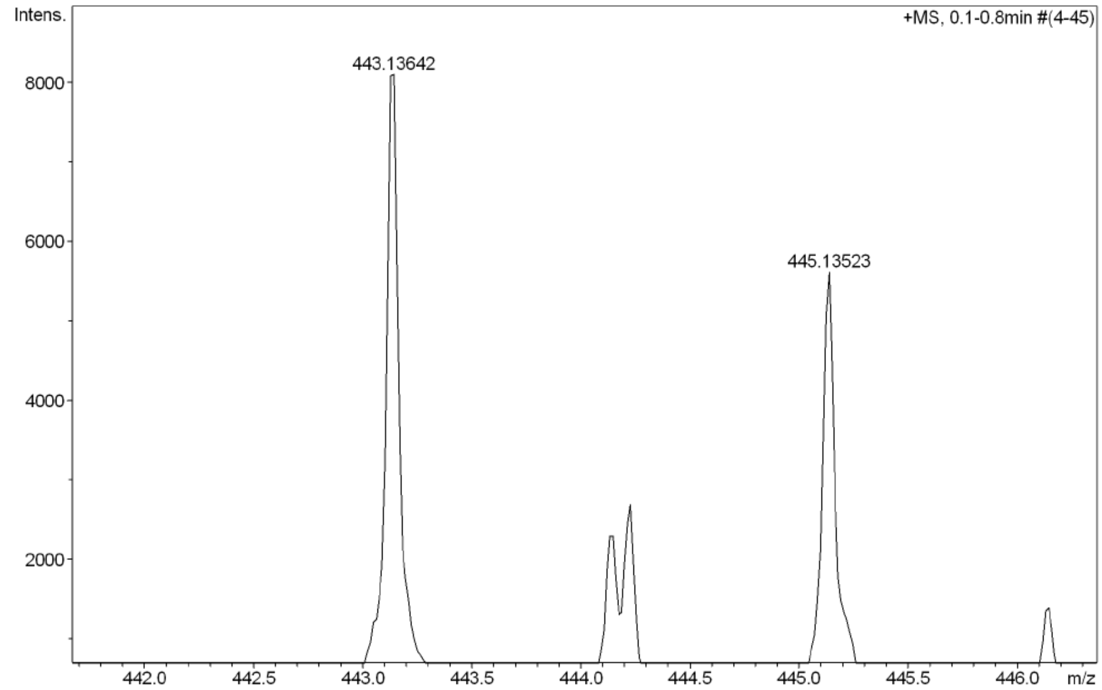

Meas. m/z	#	Formula	Score	m/z	err [mDa]	err [ppm]	mSigma	rdb	e⁻ Conf	N-Rule
443.13642	1	C 20 H 30 Cl 2 Na O 5	100.00	443.13625	-0.17	-0.39	52.2	4.5	even	ok

Figure S1. HRESIMS spectrum of **1**.

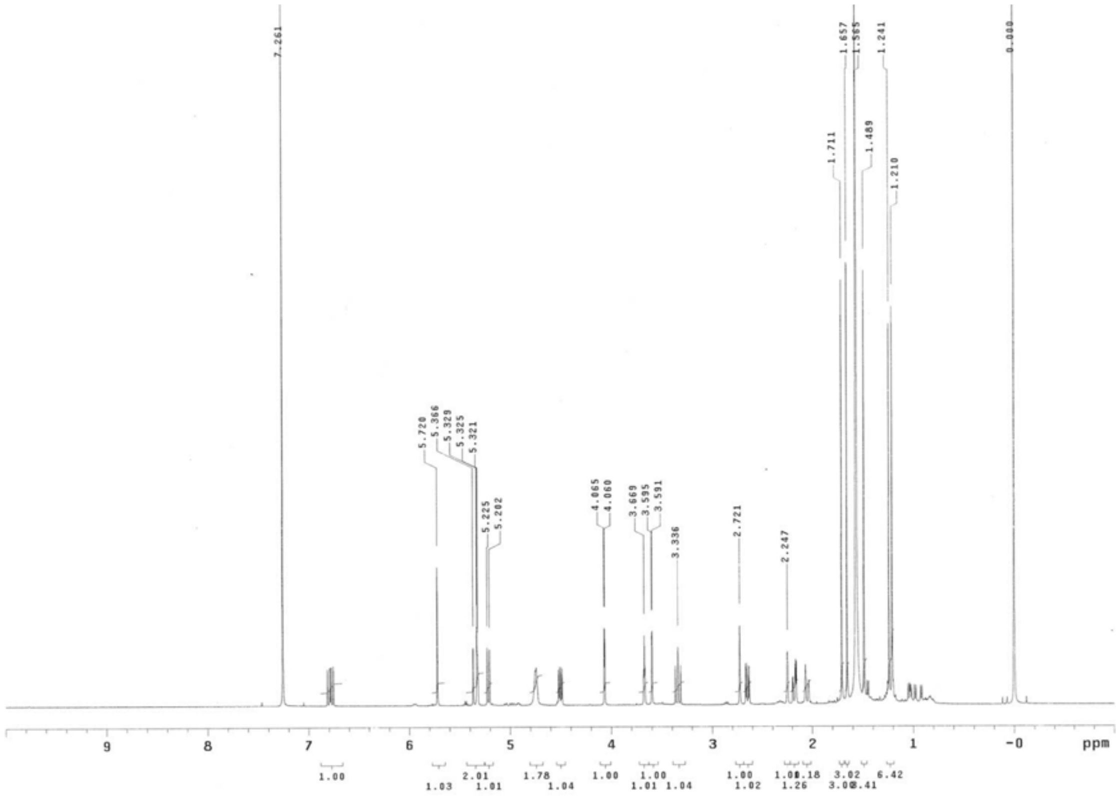

Figure S2. ^{1}H NMR spectrum of **1**.

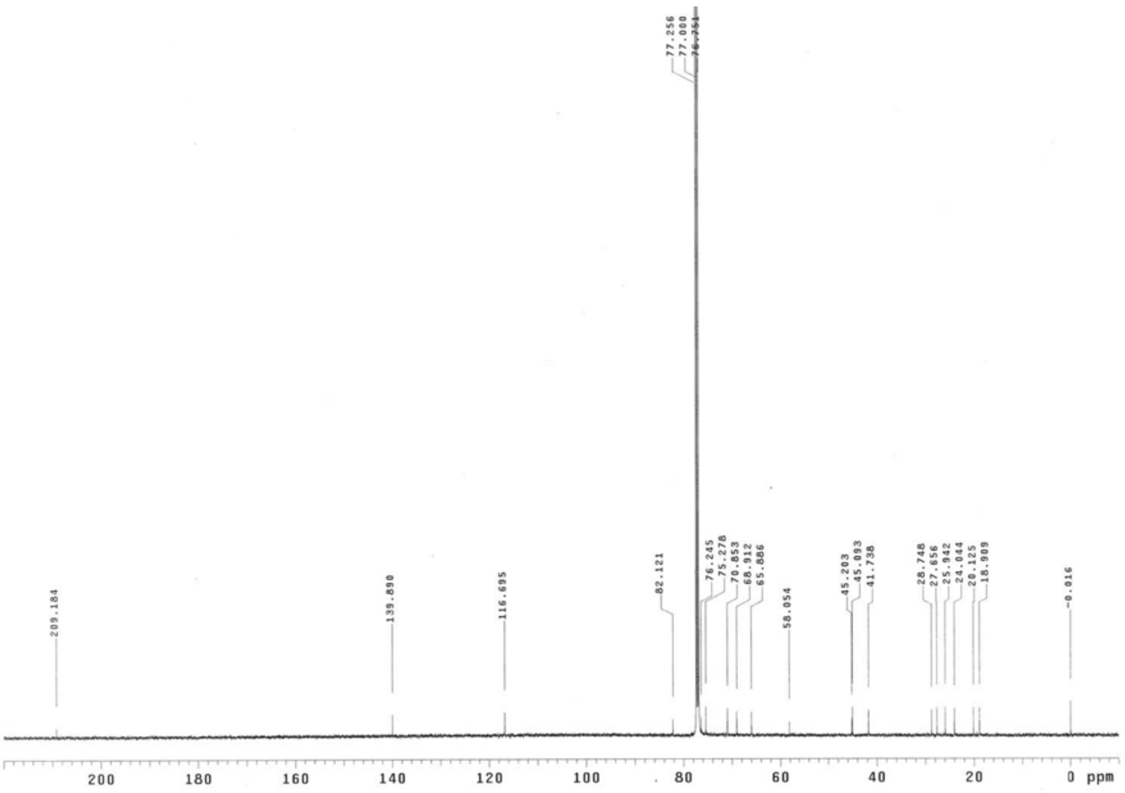

Figure S3. ^{13}C NMR spectrum of **1**.

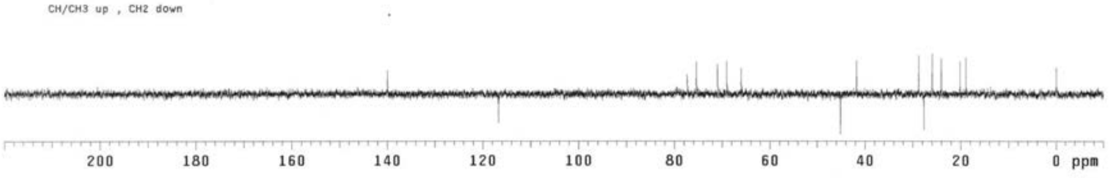

Figure S4. DEPT spectrum of **1**.

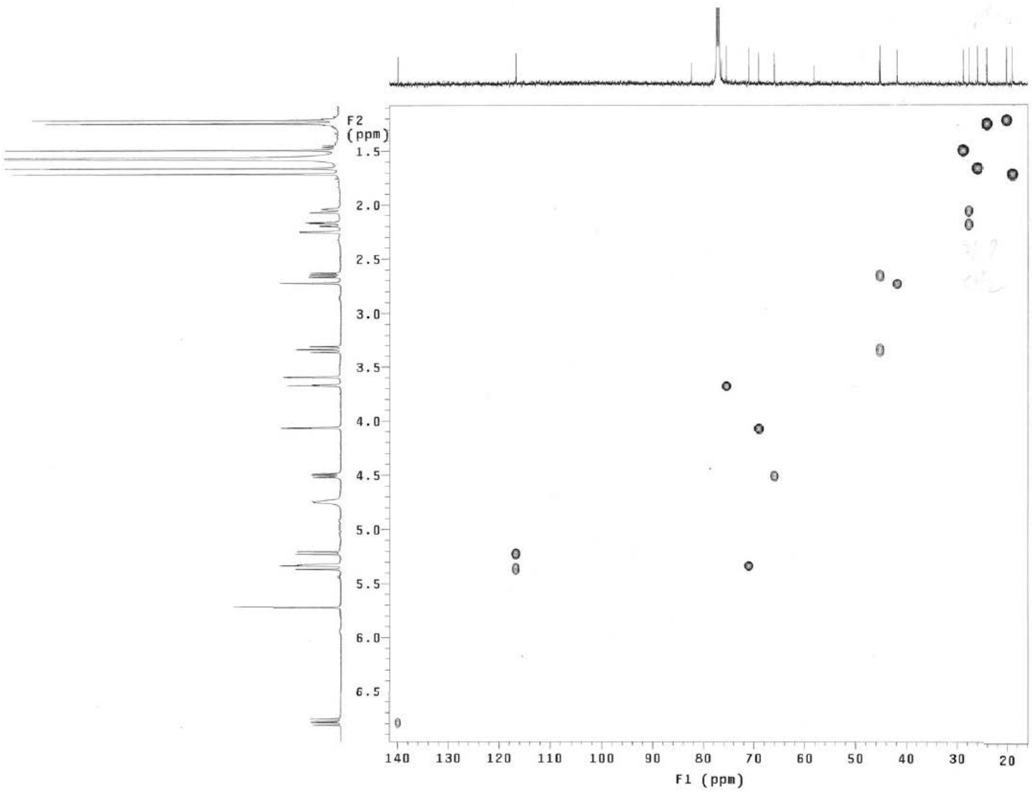

Figure S5. HSQC spectrum of **1**

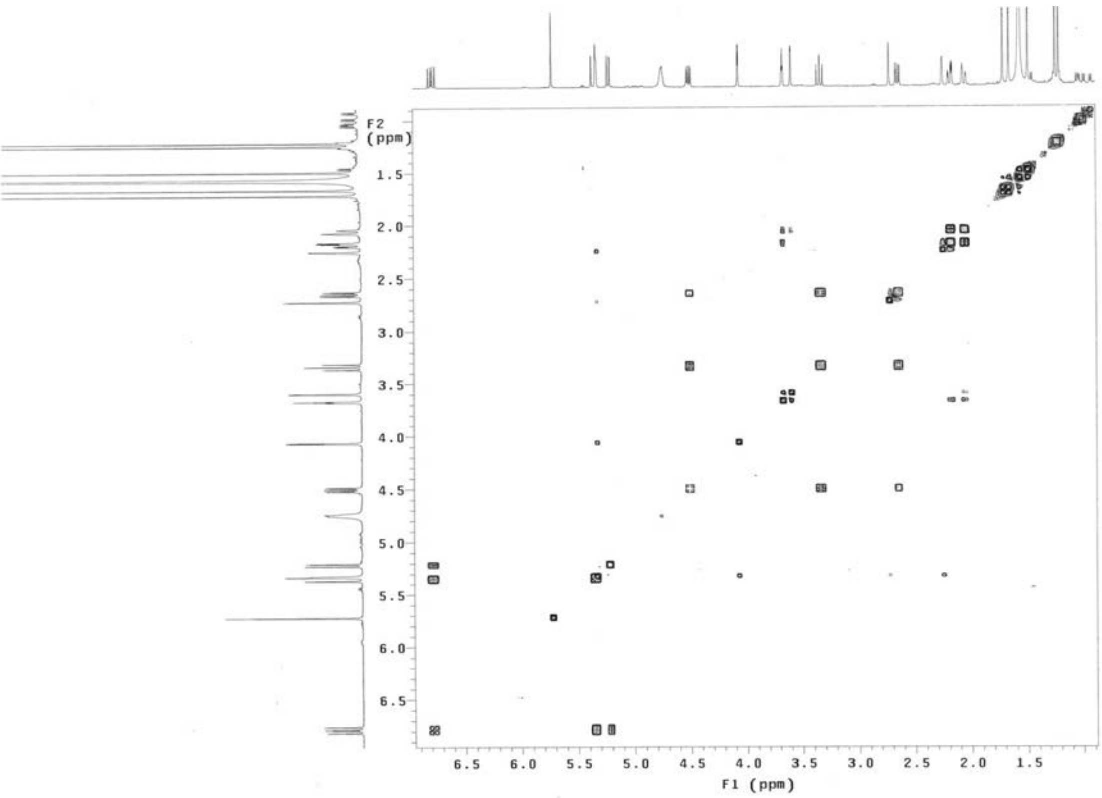

Figure S6. ^{1}H–^{1}H COSY spectrum of **1**.

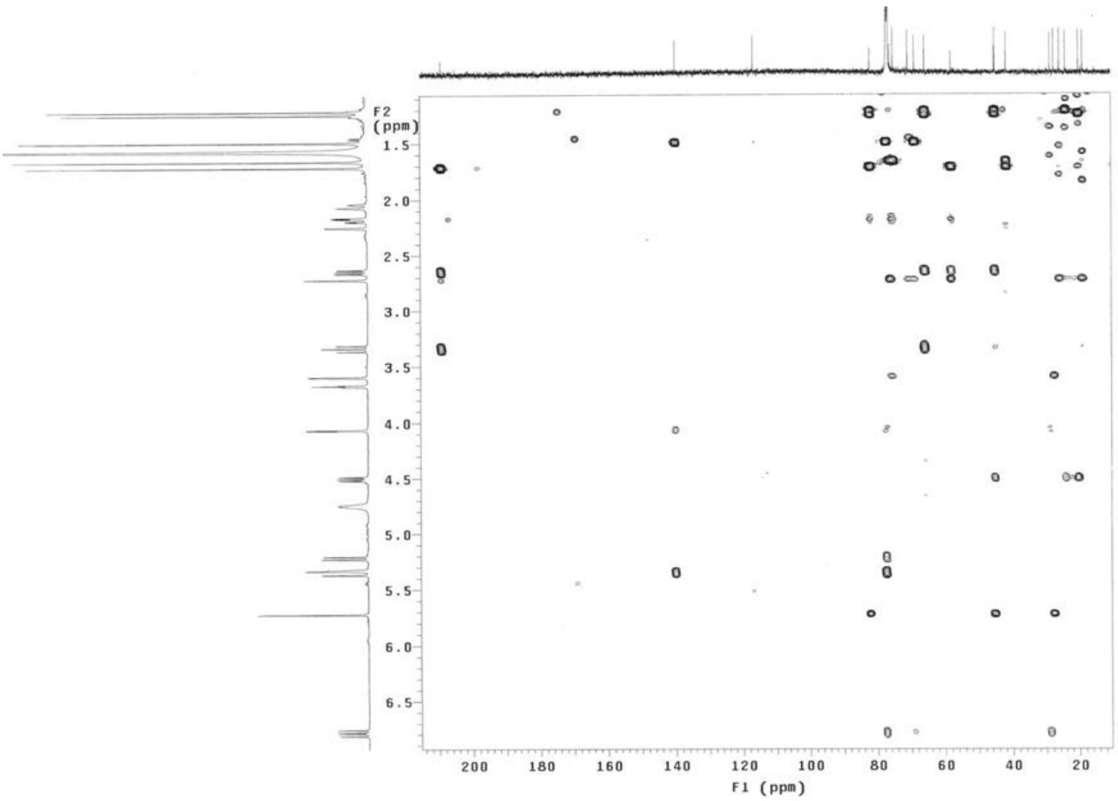

Figure S7. HMBC spectrum of **1**.

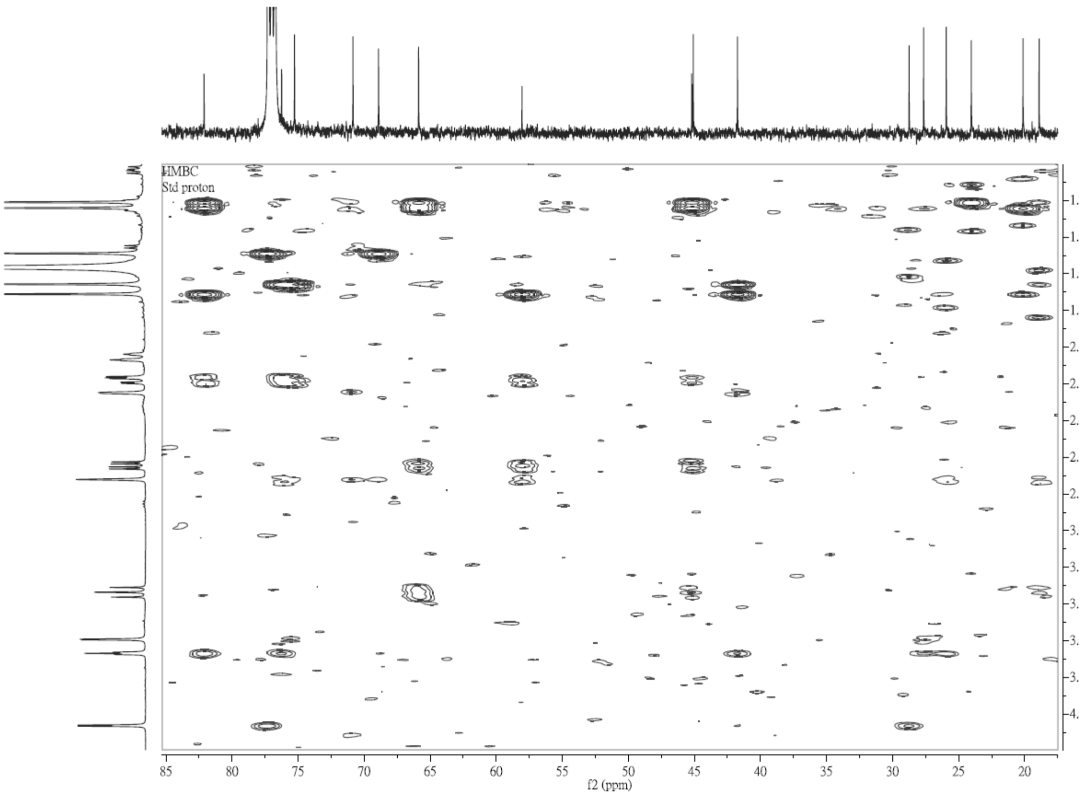

Figure S8. The partial HMBC spectrum of **1**.

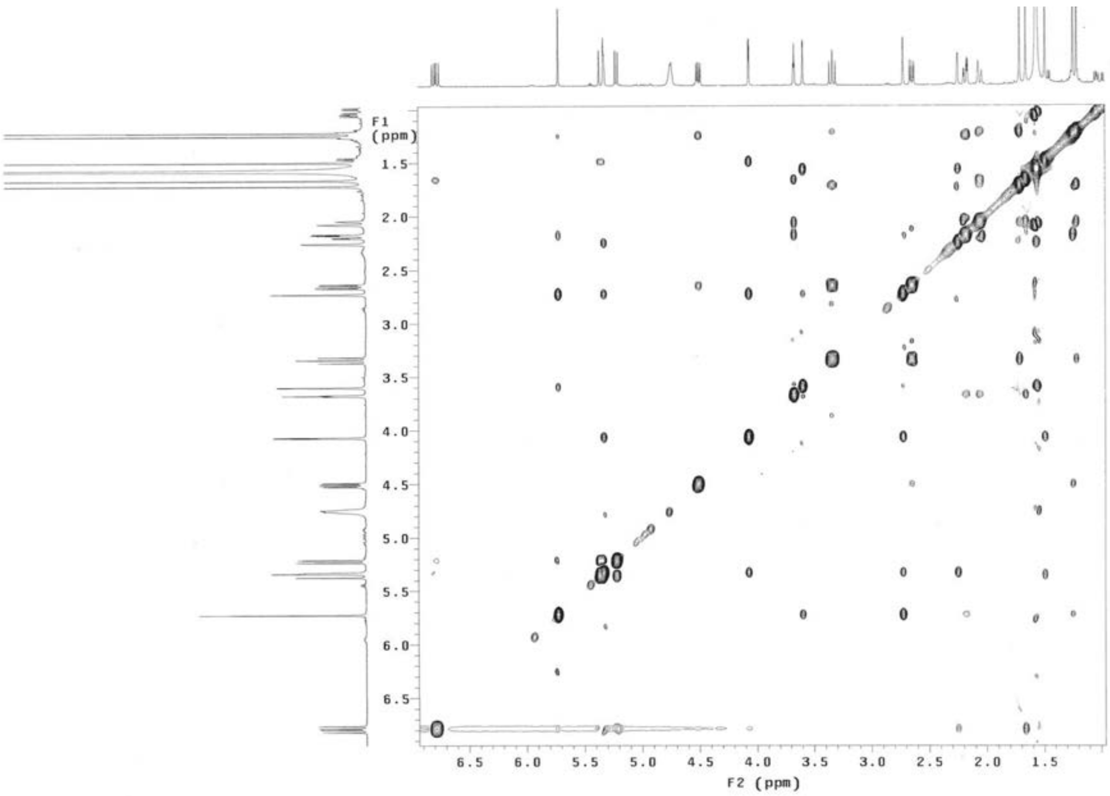

Figure S9. NOESY spectrum of **1**.

Table S5. The cartesian coordinates of conformer of compound **1** at the B3LYP/6-311+G(d,p) level of theory.

C	-2.9986360	1.8877370	-0.0588340	H	-1.2717860	-2.2681090	1.3382890
C	-3.7943200	0.6610420	-0.5062620	H	-1.7591470	-2.9274280	-0.2096550
C	-3.3435710	-0.6788630	0.1338760	H	0.6487820	-3.2629890	0.2542500
C	-1.7765200	-0.7617600	-0.1585970	H	0.5068930	0.1495050	-1.1191040
C	-0.8948330	0.4397040	0.4432860	H	2.5349820	1.0369040	-1.7287260
C	-1.4938990	1.6834840	-0.2455520	H	1.1921810	2.2557270	-0.2214640
C	-1.1849450	-2.1240730	0.2590610	H	-3.5397210	0.1184680	2.2012730
C	0.2780340	-2.3128360	-0.1543390	H	-4.8162240	-0.9733200	1.6892420
C	1.1922890	-1.1769320	0.3706160	H	-3.2346040	-1.6193630	2.1099760
C	0.5856710	0.2001460	-0.0288130	H	-5.1329310	-1.6542570	-0.6238330
C	3.4894010	-0.4005040	-0.4287980	H	-3.6990360	-1.9308070	-1.6346750
C	2.8443320	0.9877060	-0.6832990	H	-3.8998910	-2.7904210	-0.0913640
C	1.6165450	1.3357250	0.1818480	H	-0.9610090	-1.2045570	-1.8999630
O	-0.8595970	2.4765970	-0.9186200	H	-1.0266780	-0.3072980	2.5200490
C	-3.7416620	-0.7821250	1.6215800	H	-0.0961750	1.1794210	2.3240370
C	-4.0547590	-1.8375840	-0.6064030	H	-1.8527440	1.2217580	2.2843840
O	-1.6889970	-0.6228420	-1.5902820	H	1.2521800	-2.1763810	-1.8186740
C	-0.9807530	0.6416750	1.9829120	H	1.8956500	-0.5863440	2.3698540
O	0.3252970	-2.3854420	-1.5893640	H	0.5679170	-1.7439430	2.3913890
C	1.4756330	-1.4494030	1.8593440	H	2.1800200	-2.2838030	1.9230240
O	1.9441120	1.5255760	1.5582090	H	2.4776600	2.3363060	1.6134200
Cl	4.0927740	2.3301760	-0.5249340	H	3.8594310	0.1052020	1.6969080
C	4.2863140	-0.4509240	0.8693510	H	5.9148390	-1.1134010	2.0277610
C	5.4269760	-1.1162060	1.0564060	H	5.9237080	-1.6830200	0.2744800
C	4.3164920	-0.8257280	-1.6459740	H	5.1872700	-0.1779720	-1.7731310
O	2.3998580	-1.3713070	-0.4146020	H	4.6584920	-1.8573050	-1.5300960
Cl	-5.5885870	1.0222630	-0.2108500	H	3.7013190	-0.7728530	-2.5492760
H	-3.2870290	2.7628160	-0.6449270				
H	-3.2114450	2.1065750	0.9924680				
H	-3.7362000	0.5670940	-1.5887490				

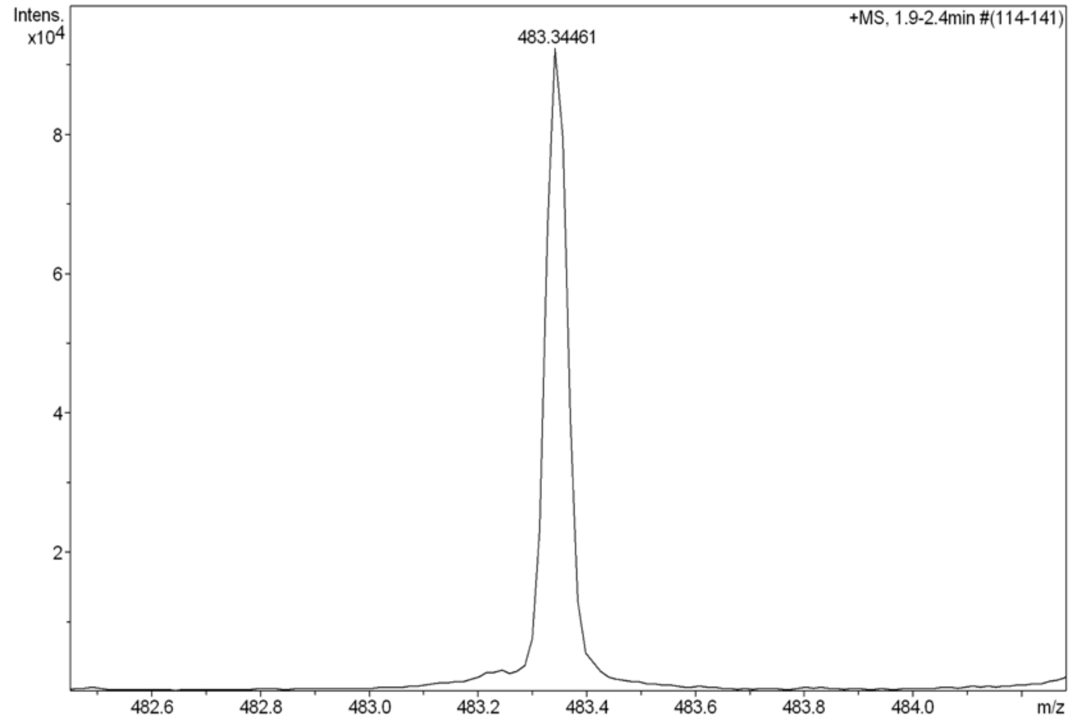

Meas. m/z	#	Formula	Score	m/z	err [mDa]	err [ppm]	mSigma	rdb	e⁻ Conf	N-Rule
483.34461	1	C 29 H 48 Na O 4	100.00	483.34448	-0.13	-0.27	22.9	5.5	even	ok

Figure S10. HRESIMS spectrum of **2**.

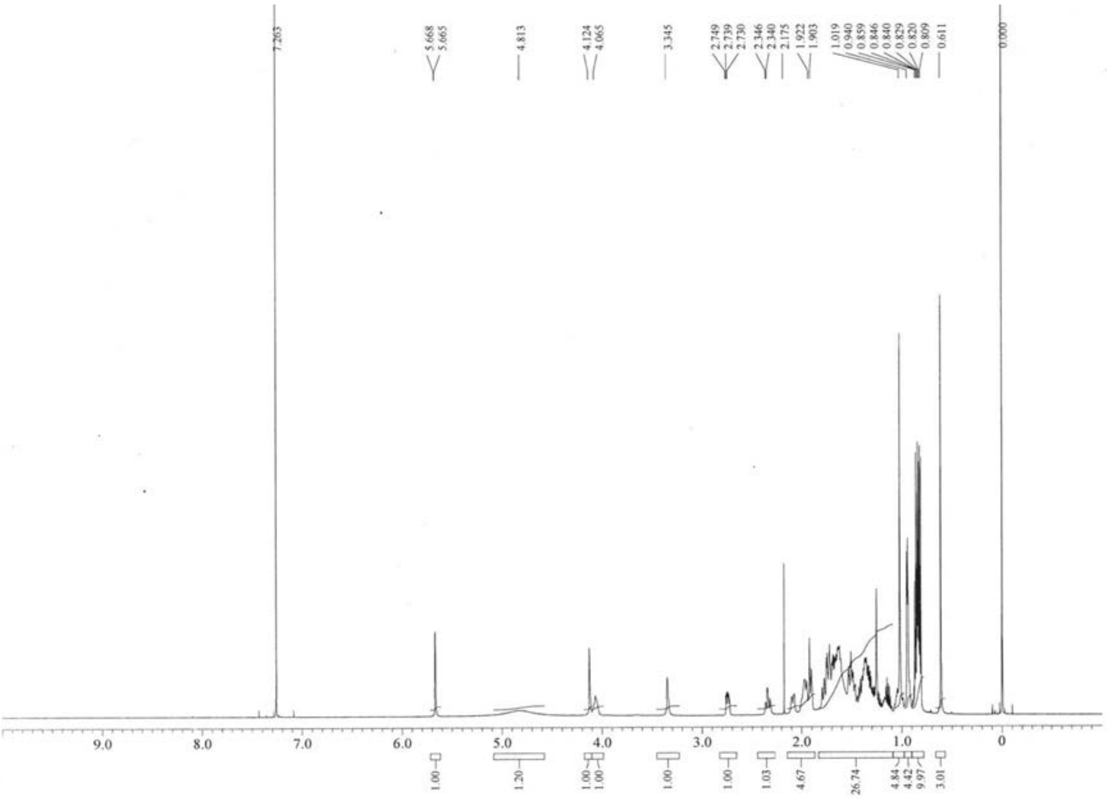

Figure S11. ^{1}H NMR spectrum of **2**.

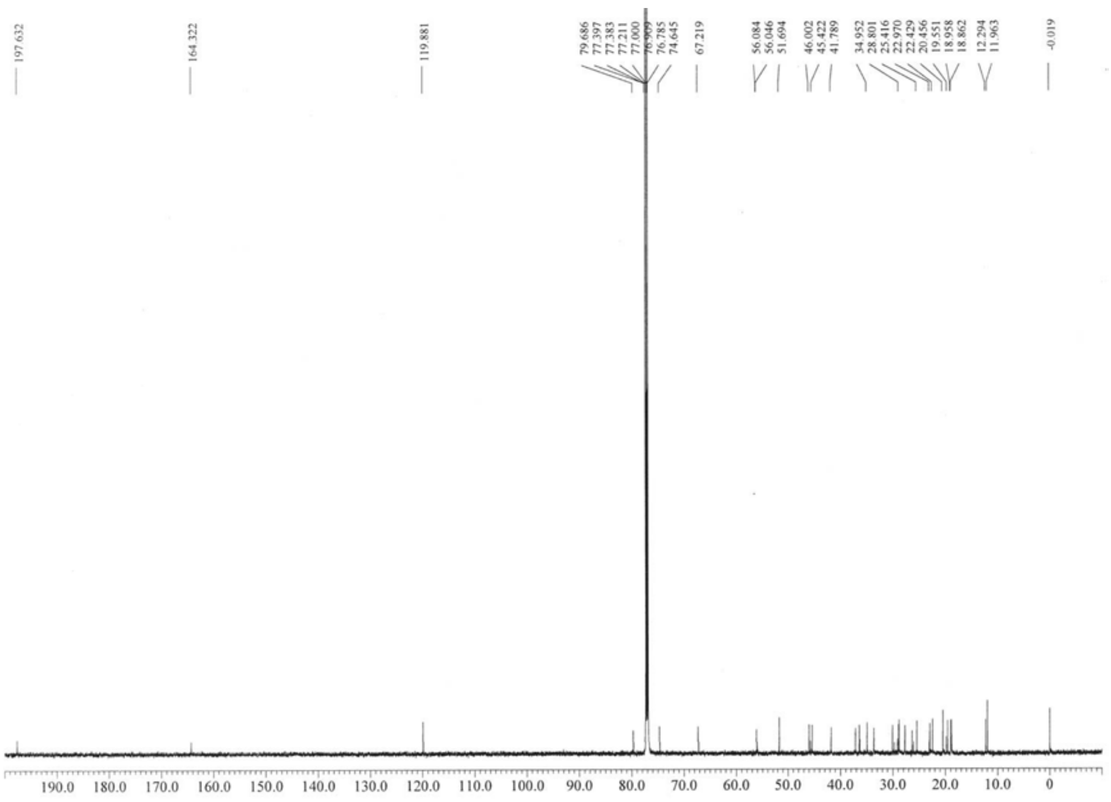

Figure S12. ^{13}C NMR spectrum of **2**.

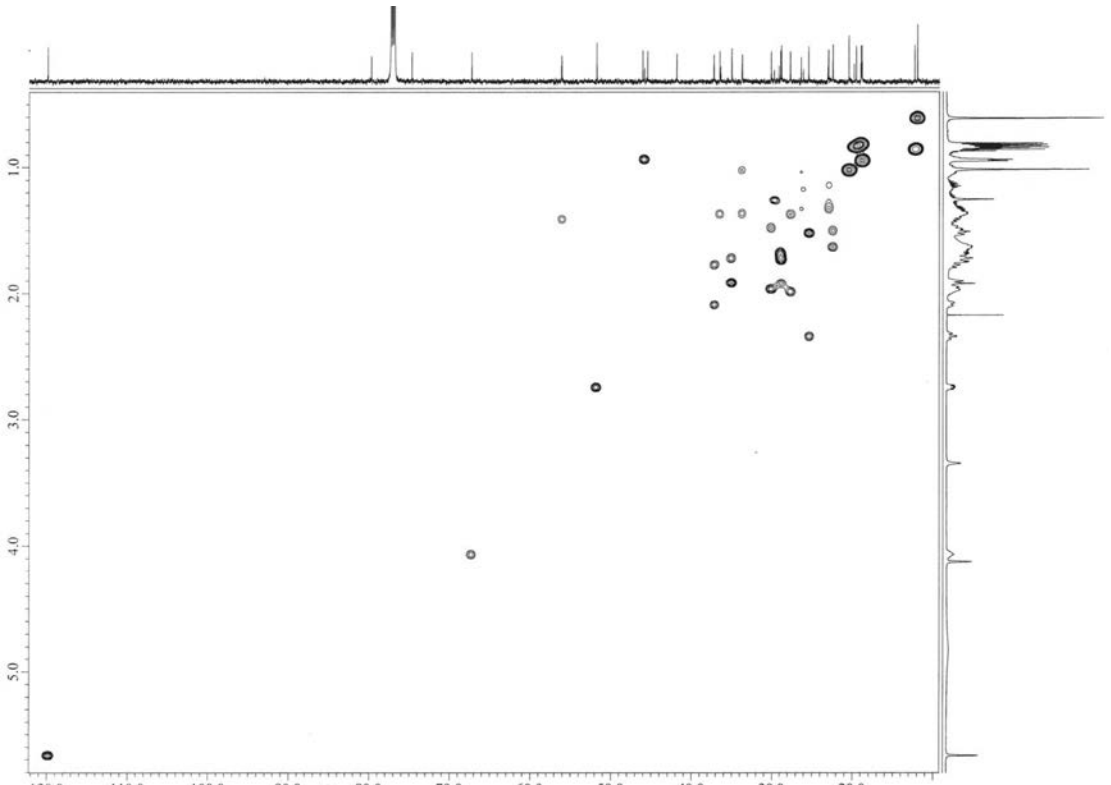

Figure S13. HSQC spectrum of **2**.

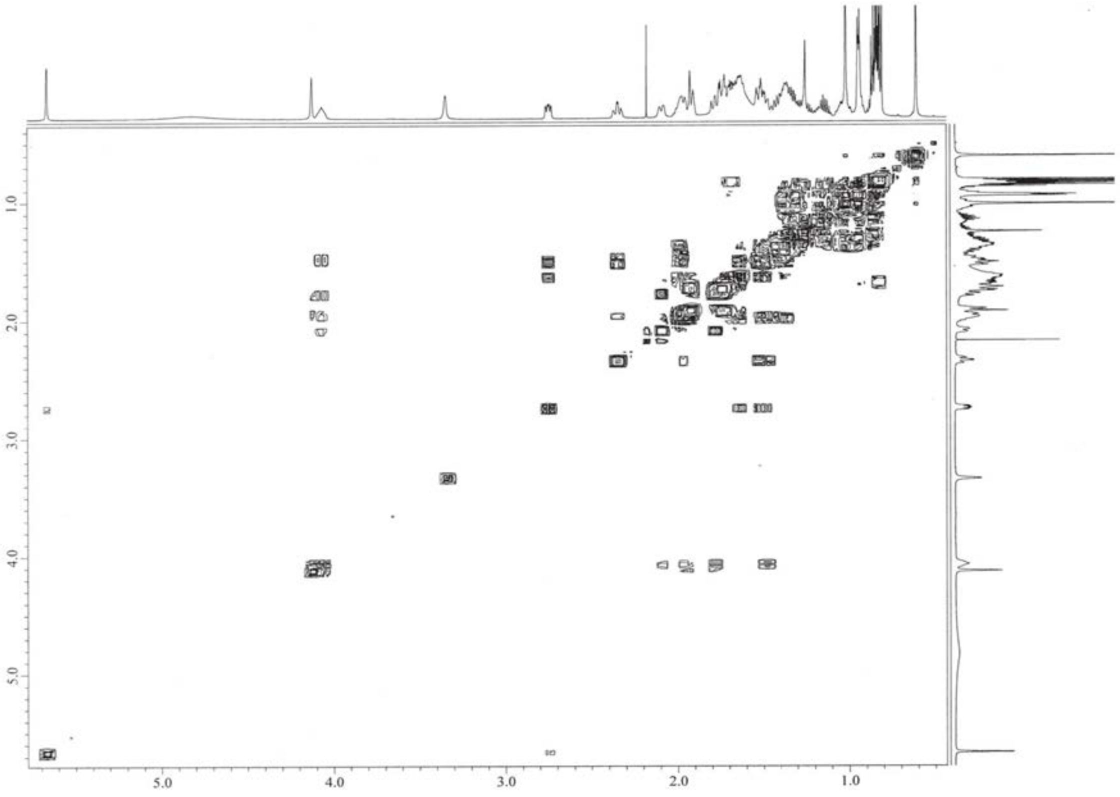

Figure S14. ^{1}H–^{1}H COSY spectrum of **2**.

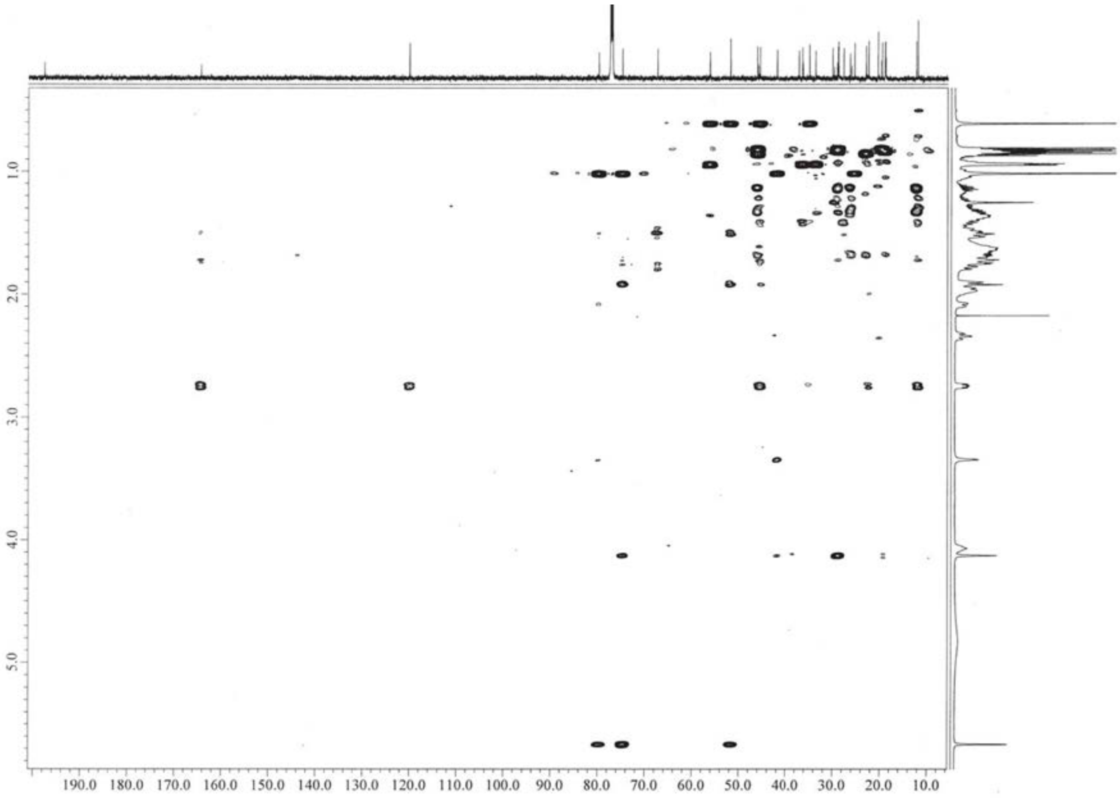

Figure S15. HMBC spectrum of **2**.

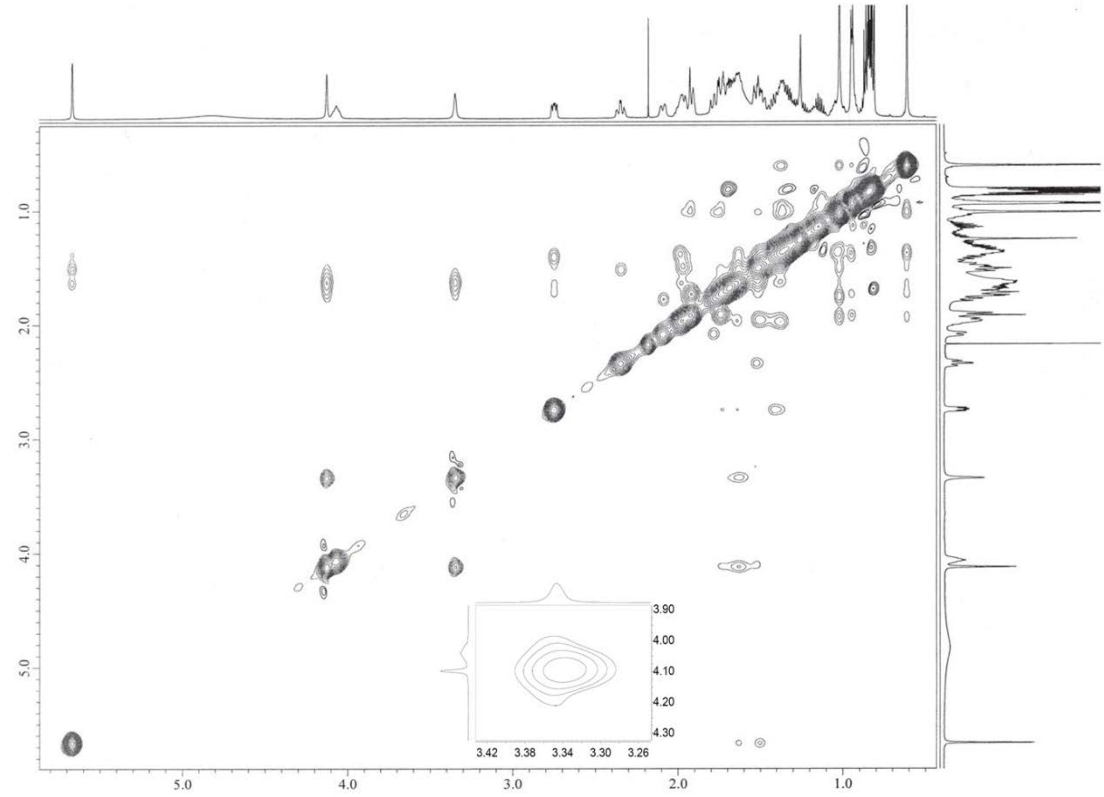

Figure S16. NOESY spectrum of **2**.

Table S6. The cartesian coordinates of conformer of compound **2** at the CAM-B3LYP/6-311+G(d,p) level of theory.

C	6.2305810	-1.6396570	0.6163380	H	7.0443720	-0.4354460	-0.9734960
C	6.9197530	-0.3659450	0.1172350	H	6.0775560	1.0369490	1.5178150
C	6.0833720	0.8735400	0.4354540	H	6.5476330	1.7652380	-0.0008870
C	4.6527430	0.7467790	-0.1018590	H	4.8416870	-1.9113270	-1.0211990
C	3.9133330	-0.5410780	0.3848590	H	4.3361900	-2.6725280	0.4762660
C	4.8022170	-1.7714030	0.0624760	H	1.8160400	2.8104100	-0.0927900
C	3.7993310	1.9943870	0.2080710	H	0.4470720	0.2183380	-1.8739620
C	2.3680720	1.8742740	-0.0745270	H	-0.3558200	-2.5972080	0.2326450
C	1.7637800	0.7018150	-0.3616830	H	0.1822250	-2.0516460	-1.3514400
C	2.5123280	-0.6476710	-0.3537570	H	1.5843870	-1.6550070	1.3361150
C	0.3468450	0.6156240	-0.8511720	H	2.1040440	-2.7180240	0.0398270
C	-0.5350890	-0.4351170	-0.1071540	H	-0.4593810	2.4618680	-0.0190630
C	0.1741860	-1.7917200	-0.2855740	H	-0.2226800	2.5279840	-1.7622000
C	1.6191700	-1.7585730	0.2498520	H	-2.3406930	1.5186580	-2.1288010
C	-0.5250940	1.8712670	-0.9398680	H	-2.6516210	1.7665400	-0.4198670
C	-1.9565030	1.3001400	-1.1279640	H	-1.7378920	-0.7330280	-1.8637430
C	-1.8821670	-0.2466420	-0.8857930	H	2.9097440	0.2620790	2.1839030
C	3.7031370	-0.4429460	1.9188000	H	3.4379350	-1.4192340	2.3330460
C	-0.7047540	-0.0792590	1.3853950	H	4.6095310	-0.1195030	2.4347230
C	-3.1950030	-0.8180830	-0.2898440	H	-1.2374510	-0.8751020	1.9173470
C	-3.1204970	-2.3251150	0.0058390	H	0.2581290	0.0626860	1.8865630
C	-4.3740290	-0.5063500	-1.2471110	H	-1.2772860	0.8438870	1.5220340
C	-5.7842360	-0.9275460	-0.7866140	H	-3.3828190	-0.2968940	0.6602140
C	-6.2862030	-0.3293570	0.5473580	H	-4.0809920	-2.7062630	0.3675820
C	-6.2972590	1.2301070	0.5650850	H	-2.8577470	-2.8921460	-0.8968100
C	-6.7589520	1.7894870	1.9210240	H	-2.3782420	-2.5567320	0.7741110
O	8.1957620	-0.1684150	0.7364680	H	-4.3826550	0.5667780	-1.4664940
O	4.3144650	3.0518070	0.5631810	H	-4.1719520	-1.0040350	-2.2079890
O	4.7190940	0.6686540	-1.5549500	H	-5.8309200	-2.0215250	-0.7047040
C	-7.5861180	-1.0383980	1.0127870	H	-6.4822810	-0.6699480	-1.5932830
C	-8.8284550	-0.9530020	0.1132270	H	-5.5457880	-0.6045360	1.3149290
C	-7.0600960	1.9179080	-0.5813810	H	-5.2443550	1.5282780	0.4531350
O	2.6782600	-1.0242650	-1.7337970	H	-7.8326900	1.6335680	2.0808980
H	6.8191310	-2.5171190	0.3163080	H	-6.5740910	2.8691580	1.9761120
H	6.2270070	-1.6245040	1.7136290	H	-6.2245750	1.3160290	2.7541620

H	8.7503770	-0.9293080	0.4995580
H	5.1537180	1.4722370	-1.8872640
H	-7.8518300	-0.6802450	2.0152950
H	-7.3339510	-2.1008830	1.1403500
H	-9.6138730	-1.6163450	0.4951960
H	-8.6128970	-1.2607870	-0.9160140
H	-9.2478760	0.0572560	0.0774890
H	-6.8922180	3.0013980	-0.5418690
H	-8.1405780	1.7547490	-0.5157230
H	-6.7297230	1.5722150	-1.5667160
H	3.3211070	-0.3840290	-2.0991460

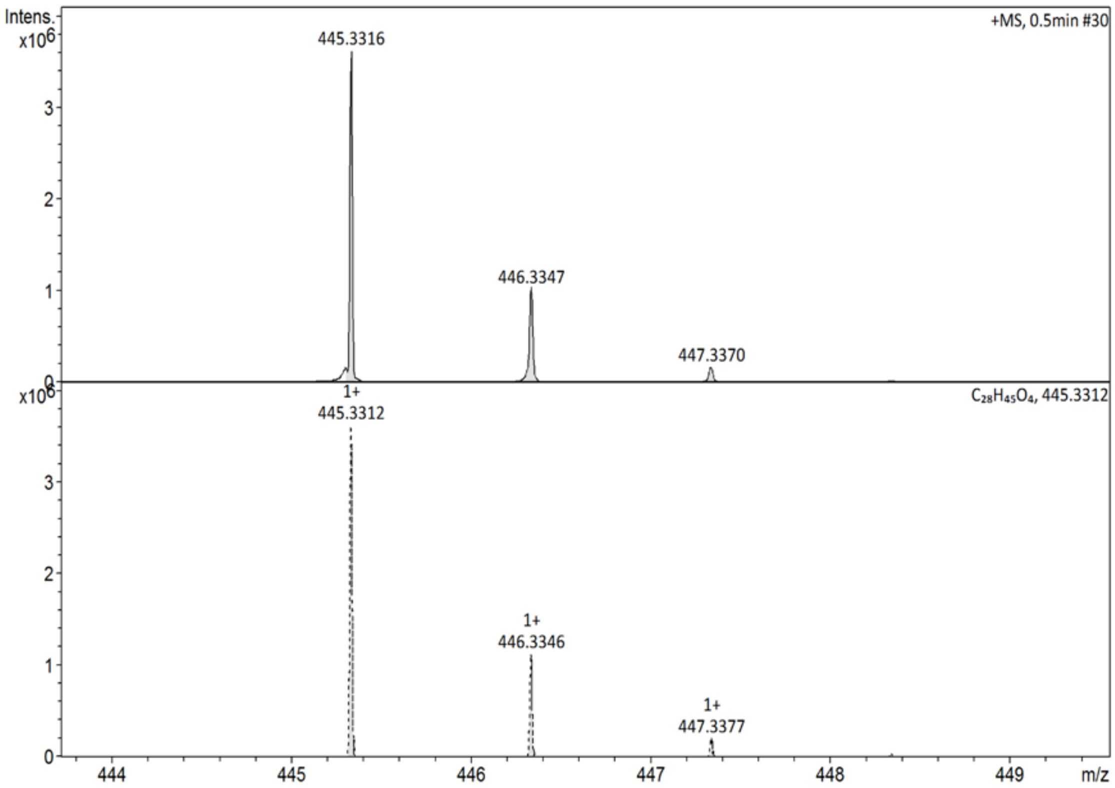

Figure S17. HRESIMS spectrum of **3**.

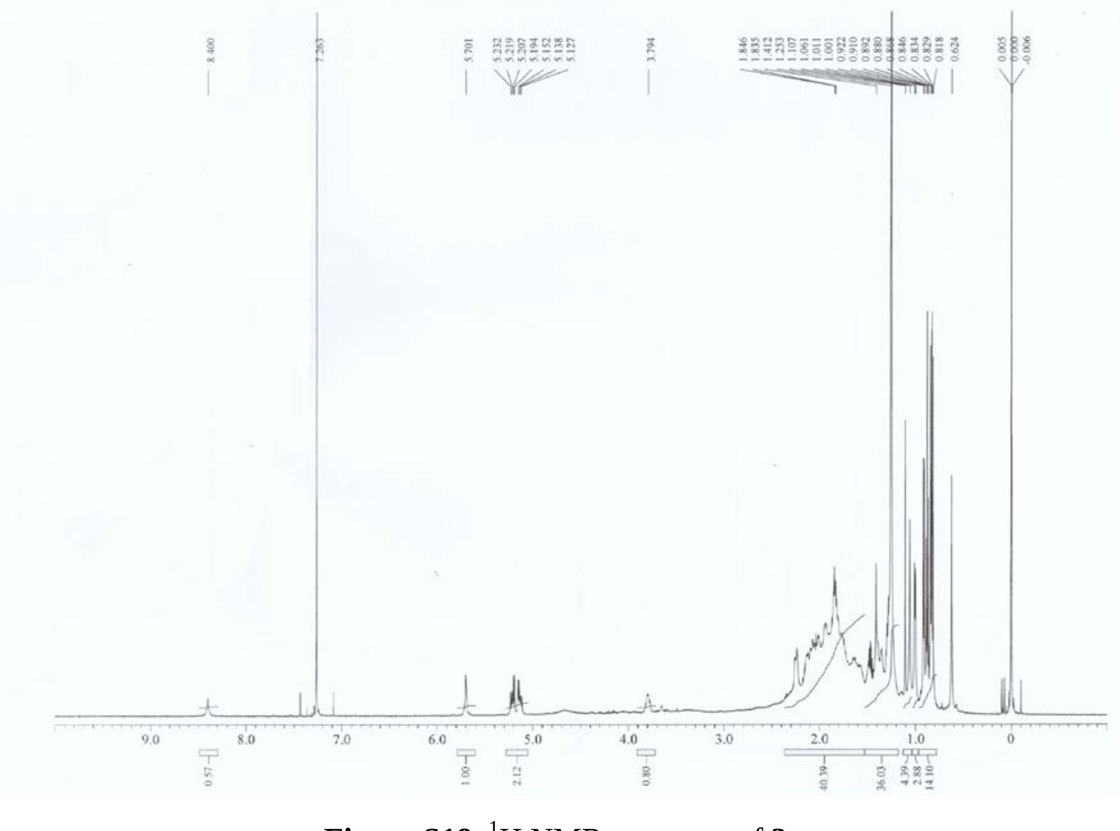

Figure S18. ^{1}H NMR spectrum of **3**.

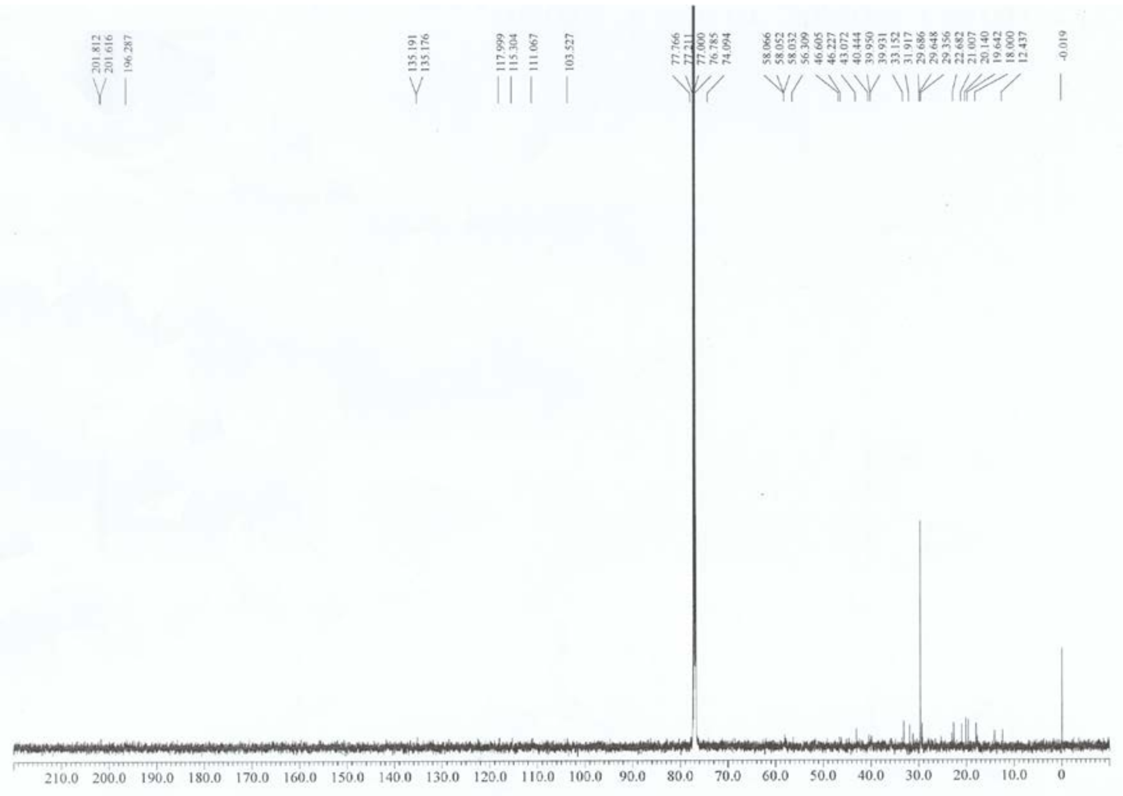

Figure S19. ^{13}C NMR spectrum of **3**.

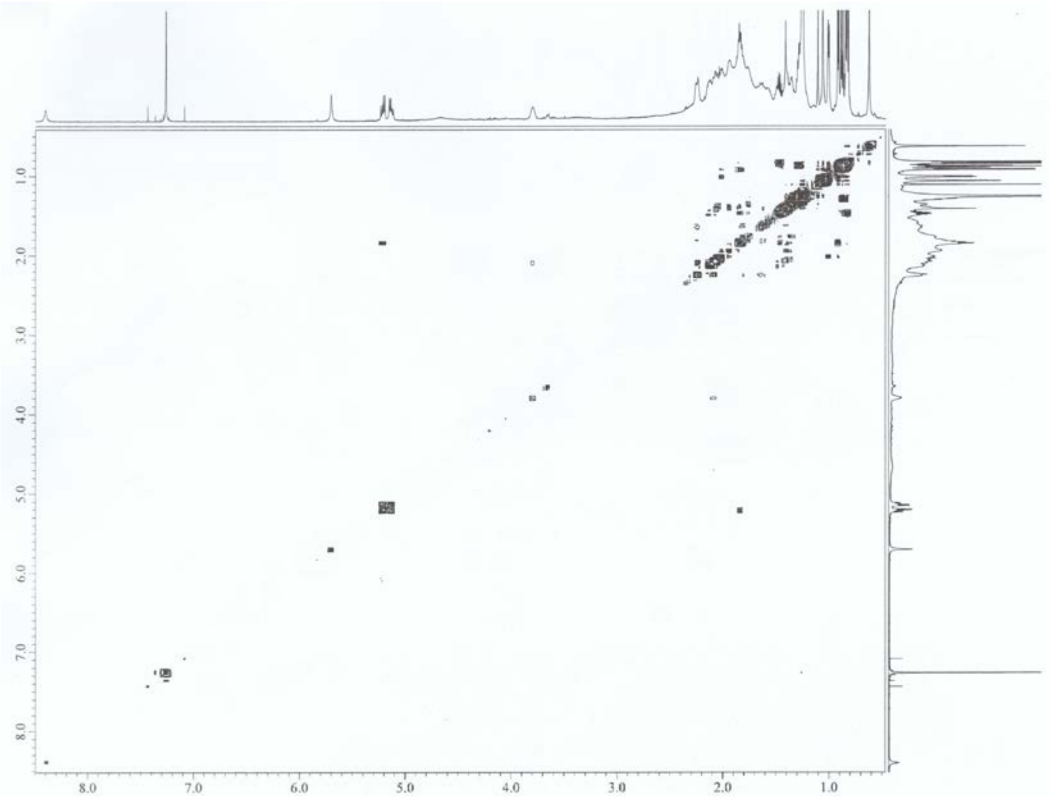

Figure S20. ^{1}H–^{1}H COSY spectrum of **3**.

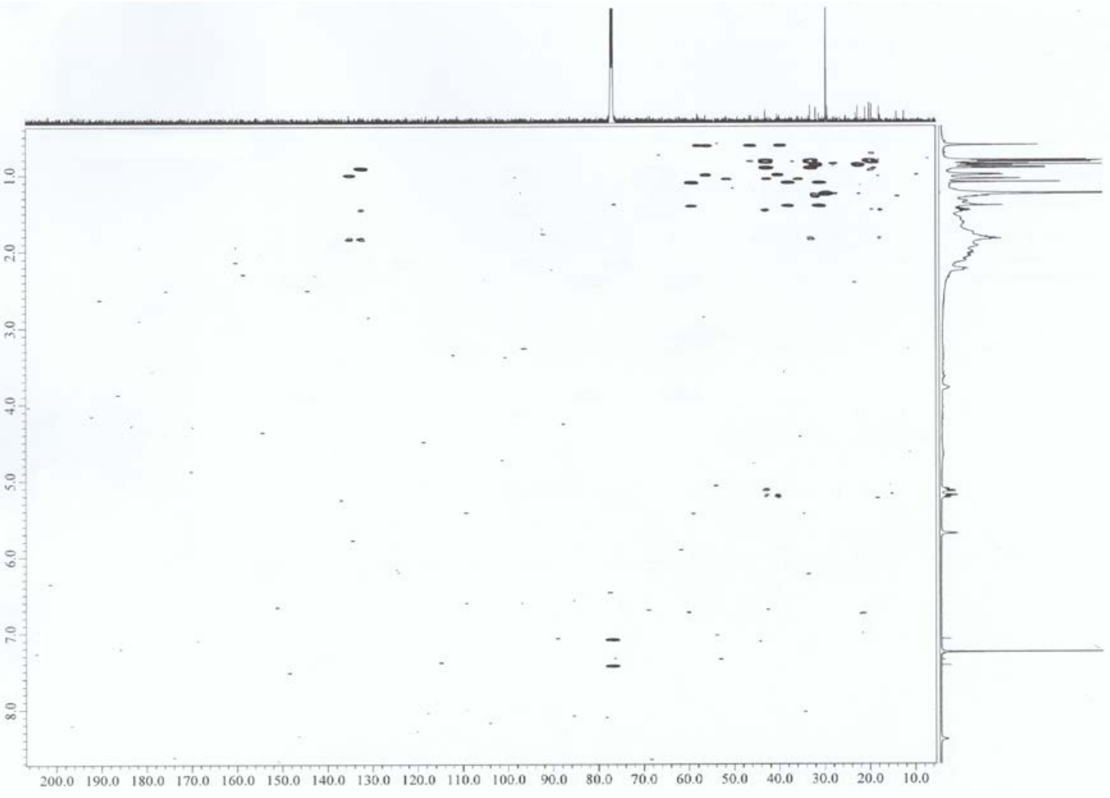

Figure S21. HMBC spectrum of **3**.

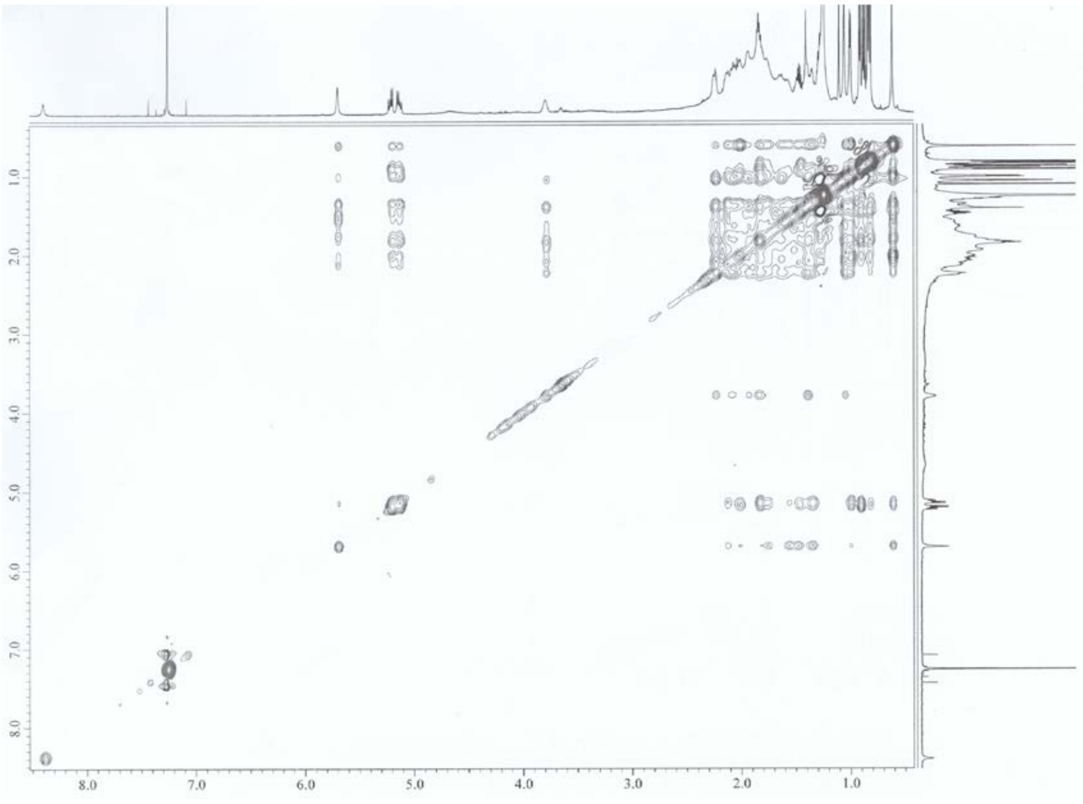

Figure S22. NOESY spectrum of **3**.

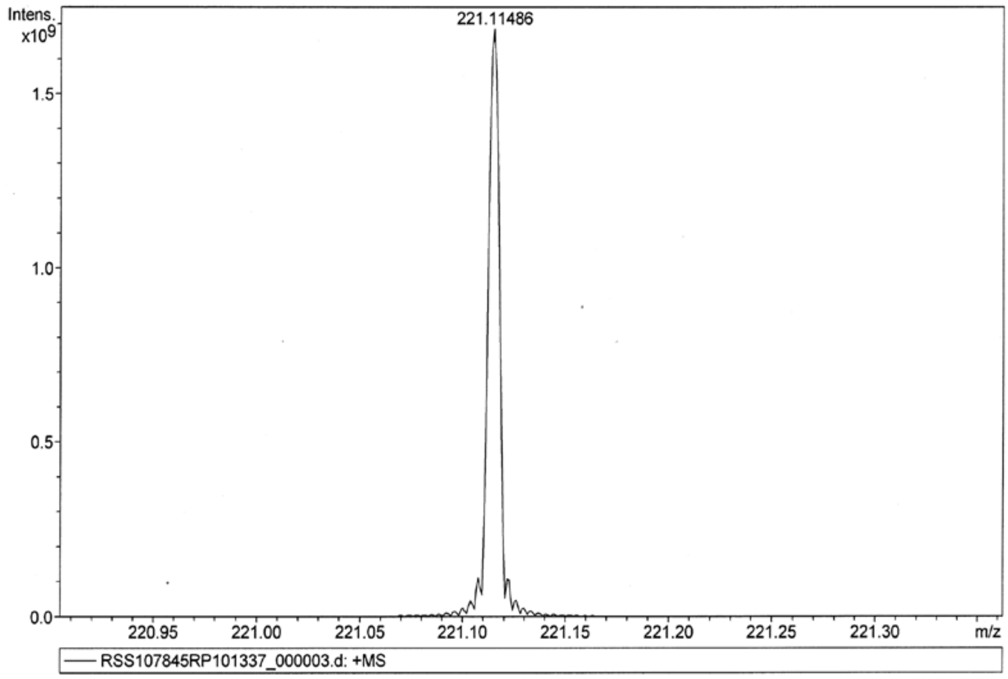

Meas. m/z	#	Formula	Score	m/z	err [mDa]	err [ppm]	mSigma	rdb	e⁻ Conf	N-Rule
221.11486	1	C 11 H 18 Na O 3	100.00	221.11482	-0.05	-0.22	6.3	2.5	even	ok

Figure S23. HRESIMS spectrum of **4**.

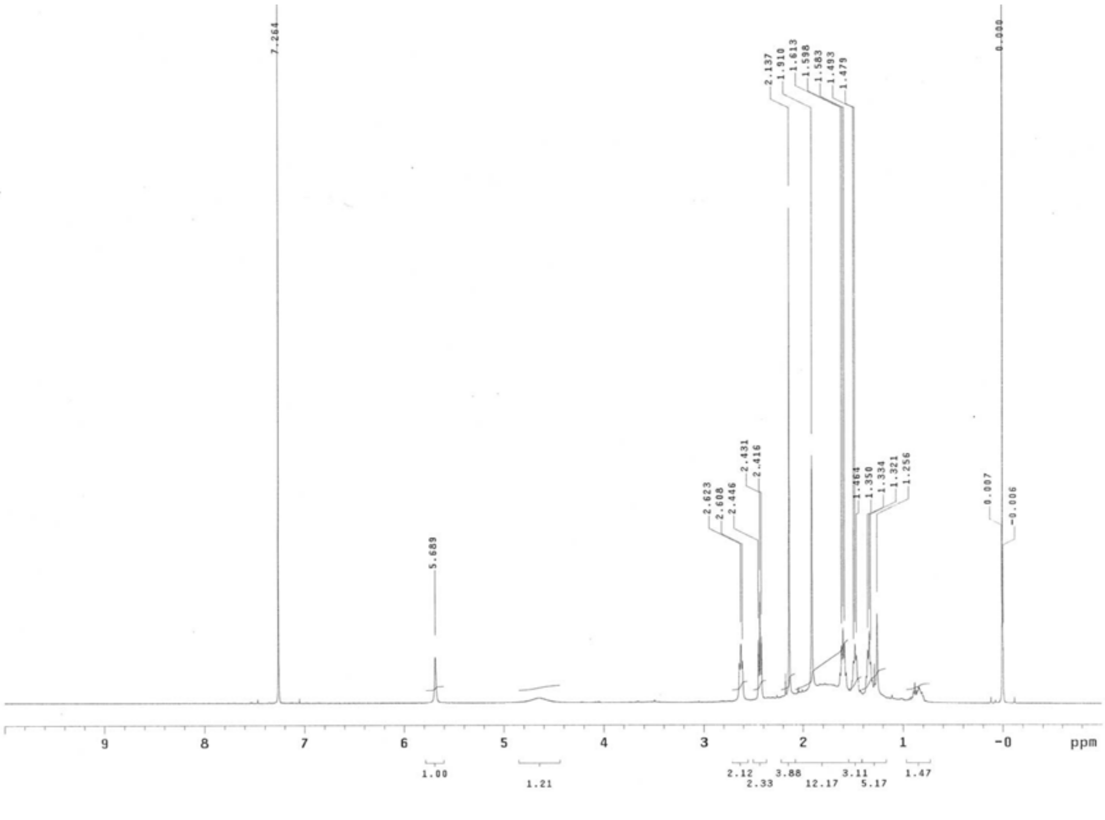

Figure S24. ^{1}H NMR spectrum of **4**.

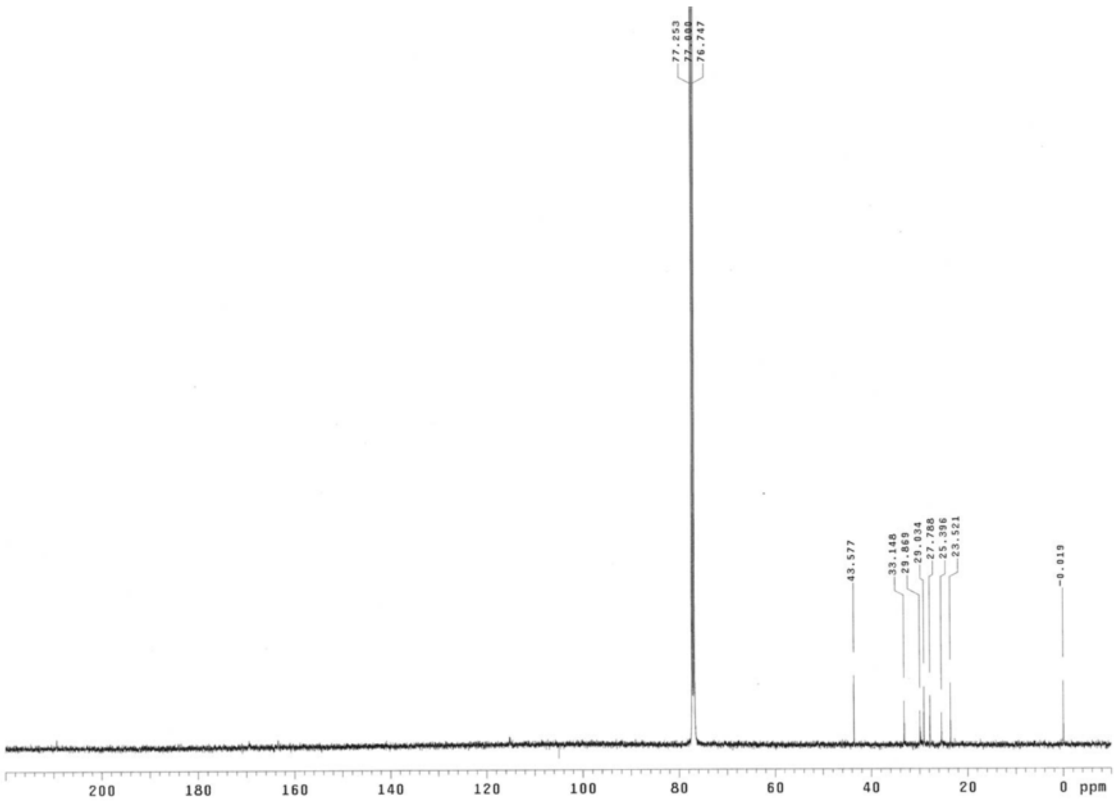

Figure S25. ^{13}C NMR spectrum of **4**.

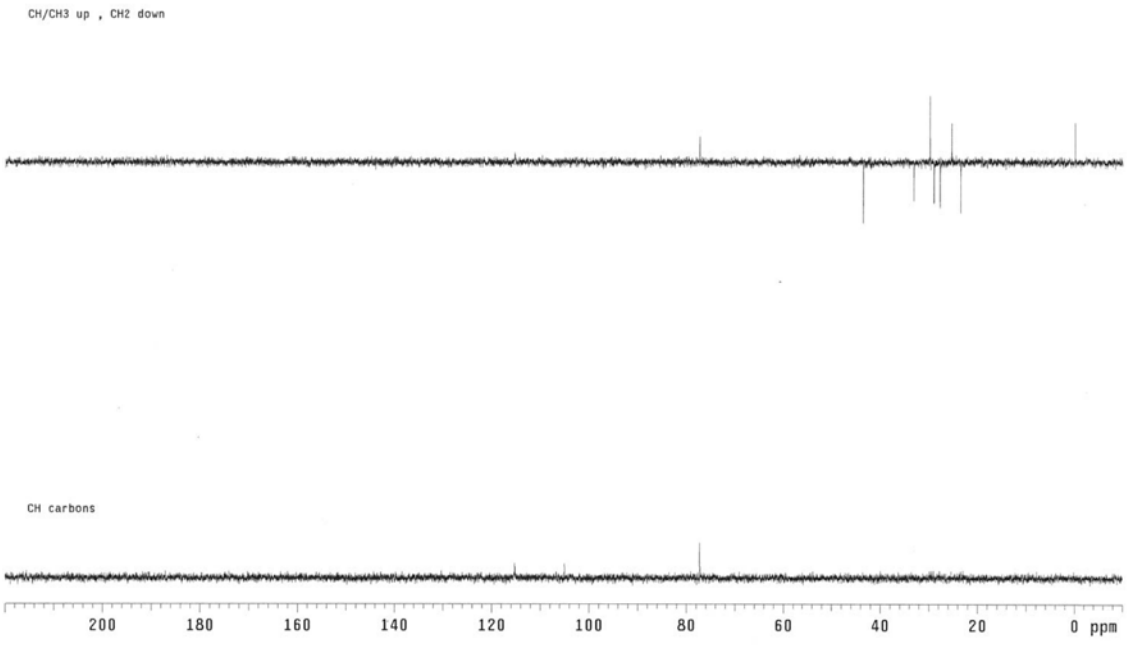

Figure S26. DEPT spectrum of **4**.

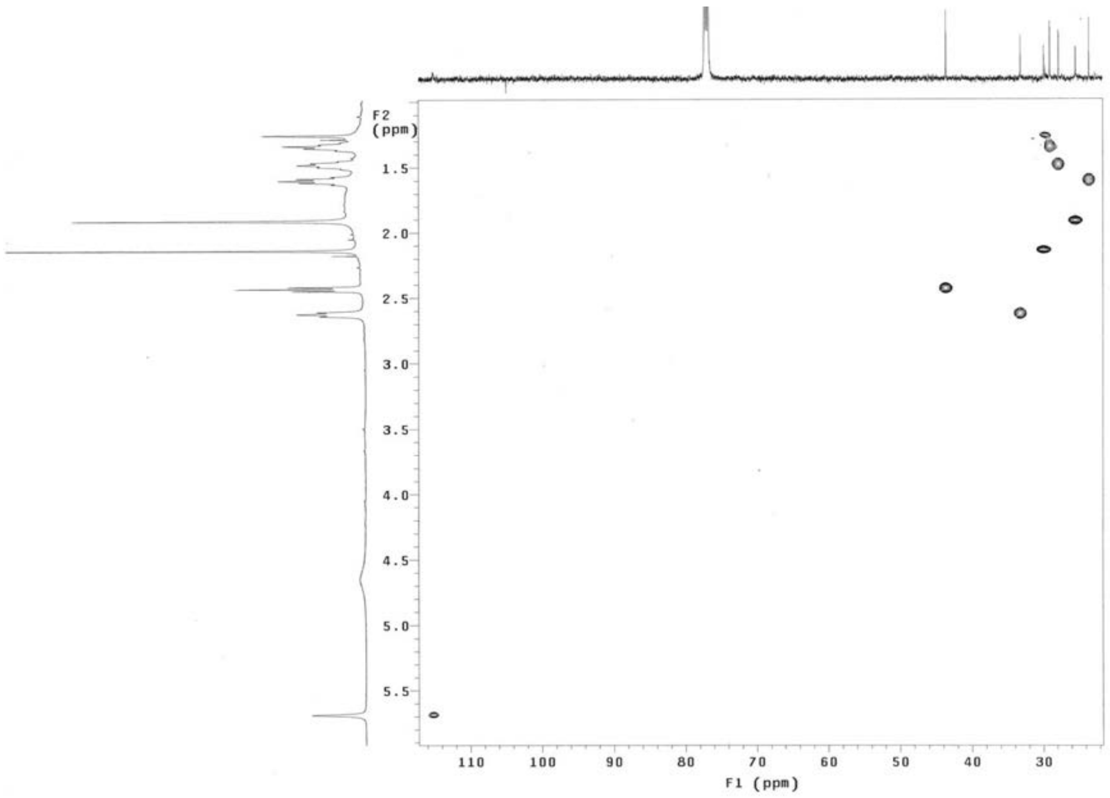

Figure S27. HSQC spectrum of **4**.

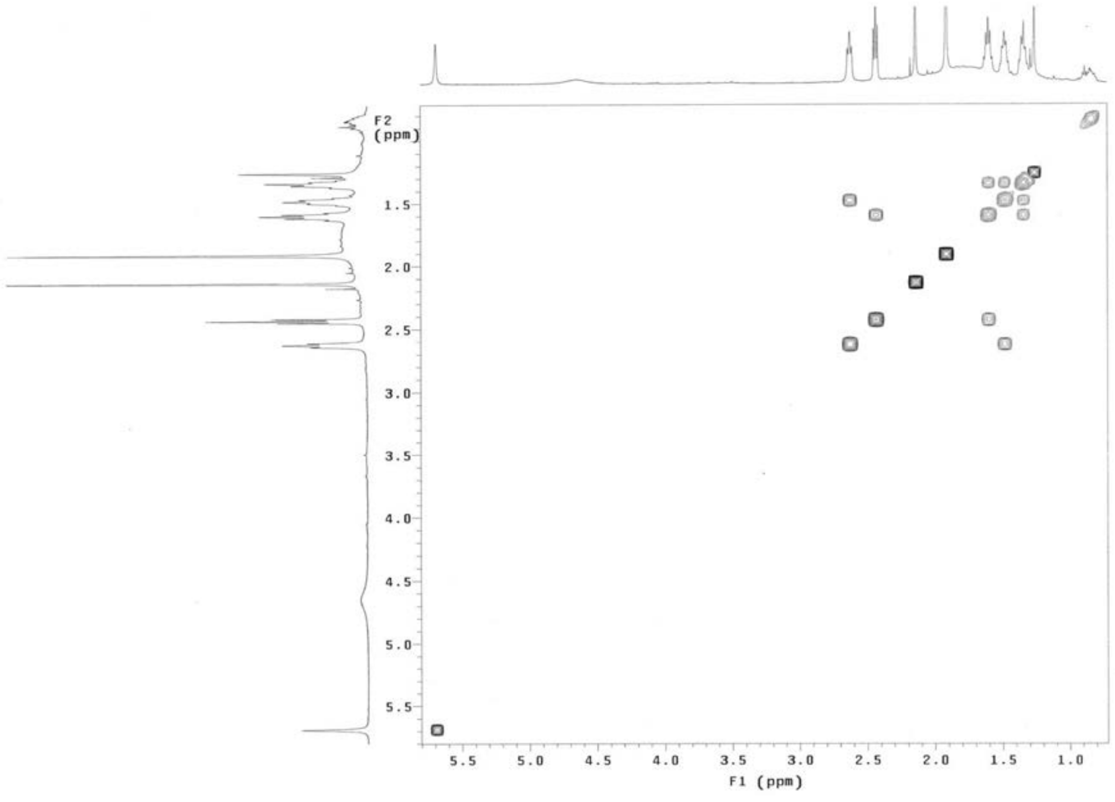

Figure S28. ^{1}H–^{1}H COSY spectrum of **4**.

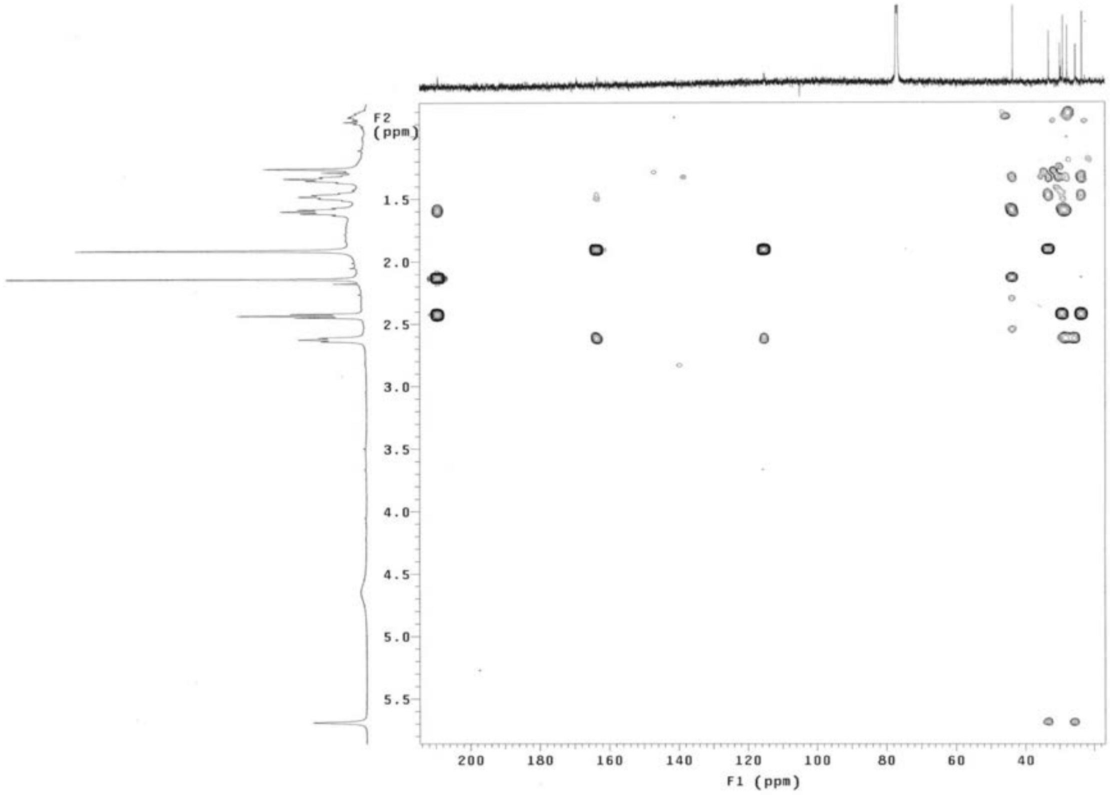

Figure S29. HMBC spectrum of **4**.

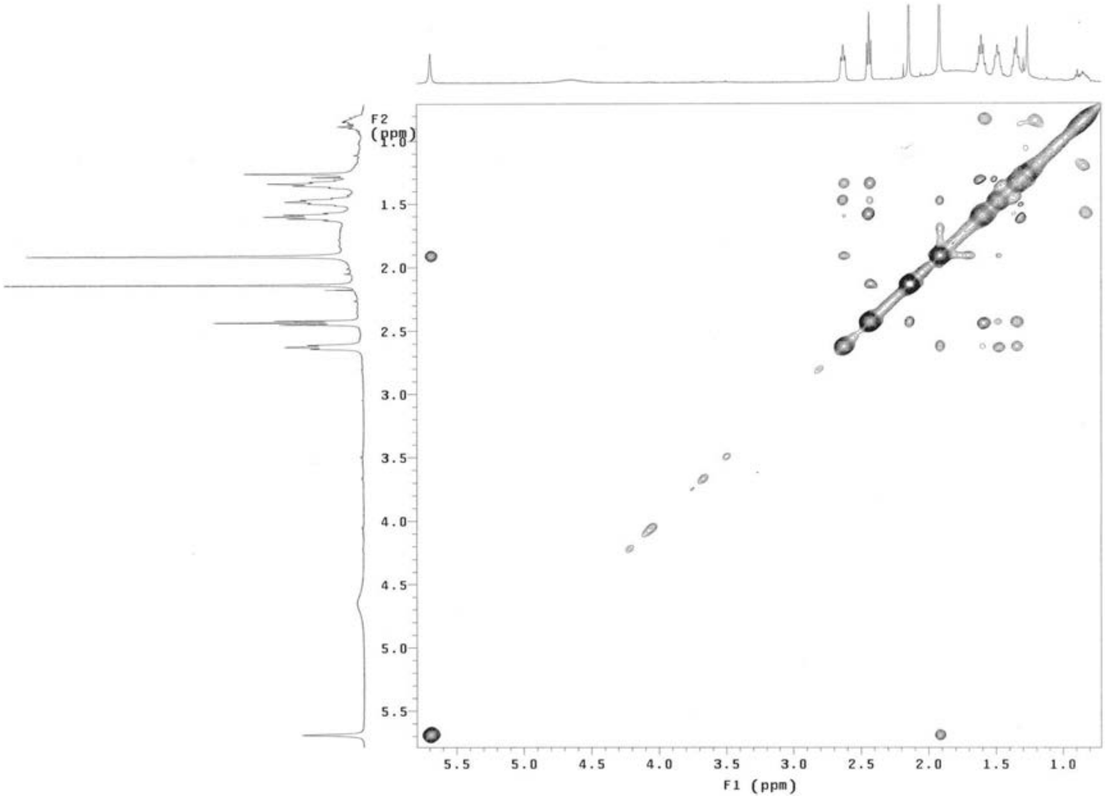

Figure S30. NOESY spectrum of **4**.

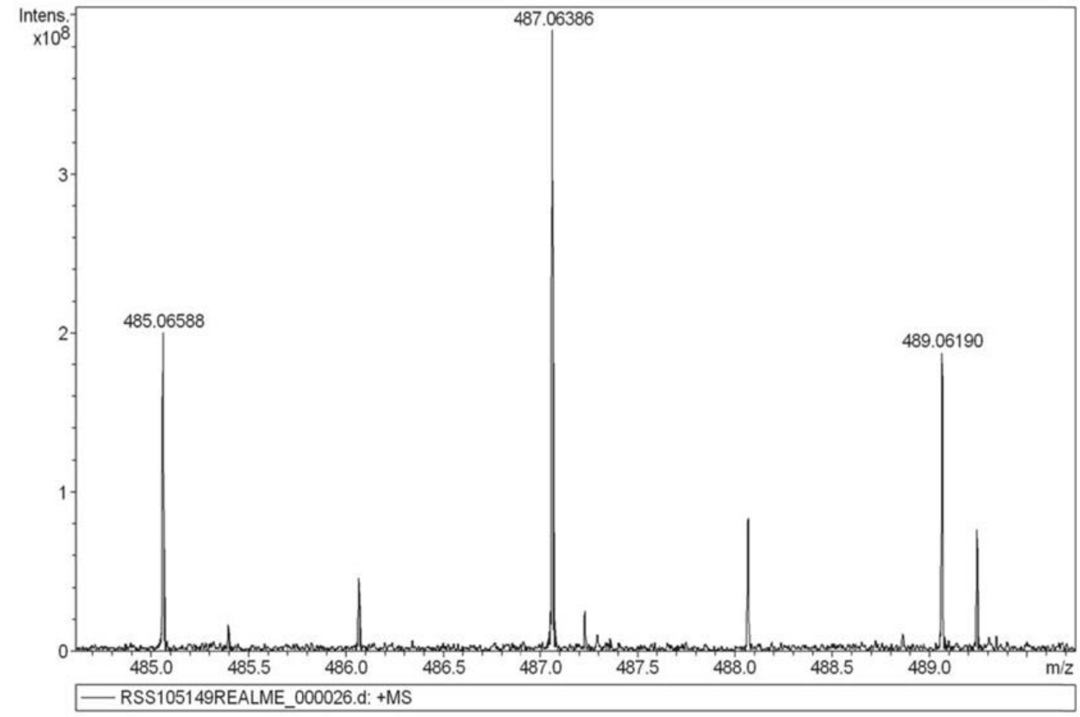

Meas. m/z	#	Formula	Score	m/z	err [mDa]	err [ppm]	mSigma	rdb	e^- Conf	N-Rule
485.06588	1	C 20 H 32 Br 2 Na O 2	100.00	485.06613	0.25	0.51	11.5	3.5	even	ok

Figure S31. HRESIMS spectrum of **5**.

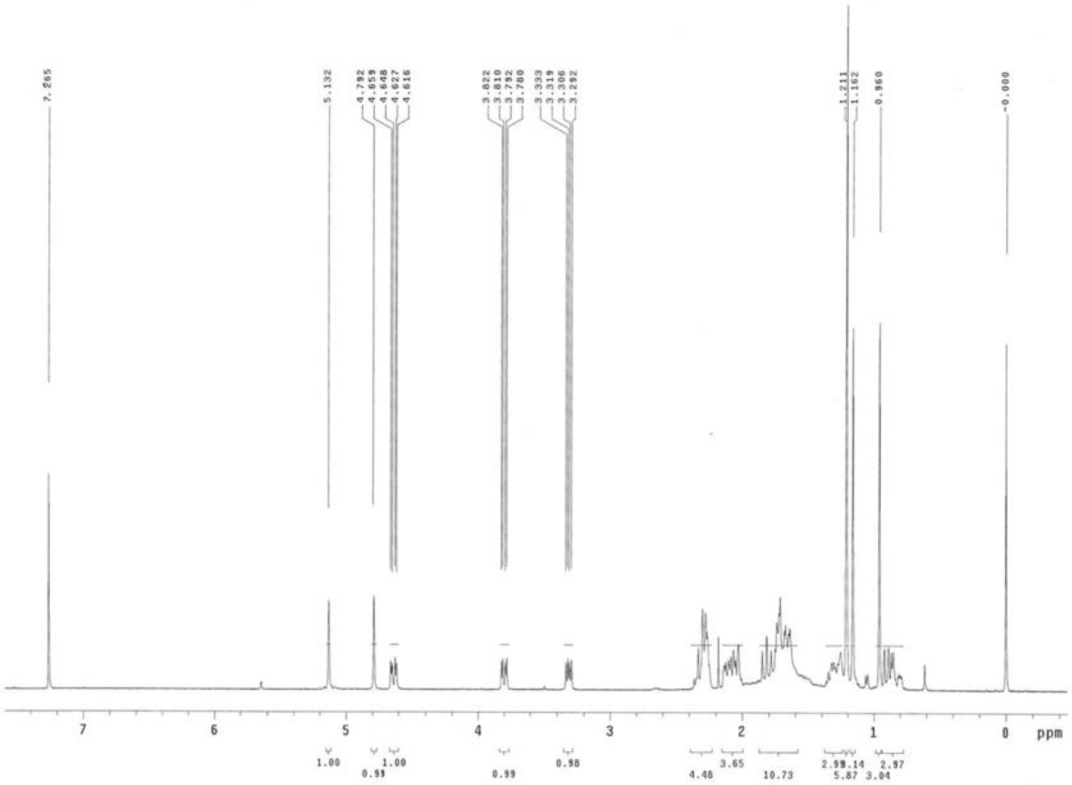

Figure S32. ^{1}H NMR spectrum of **5**.

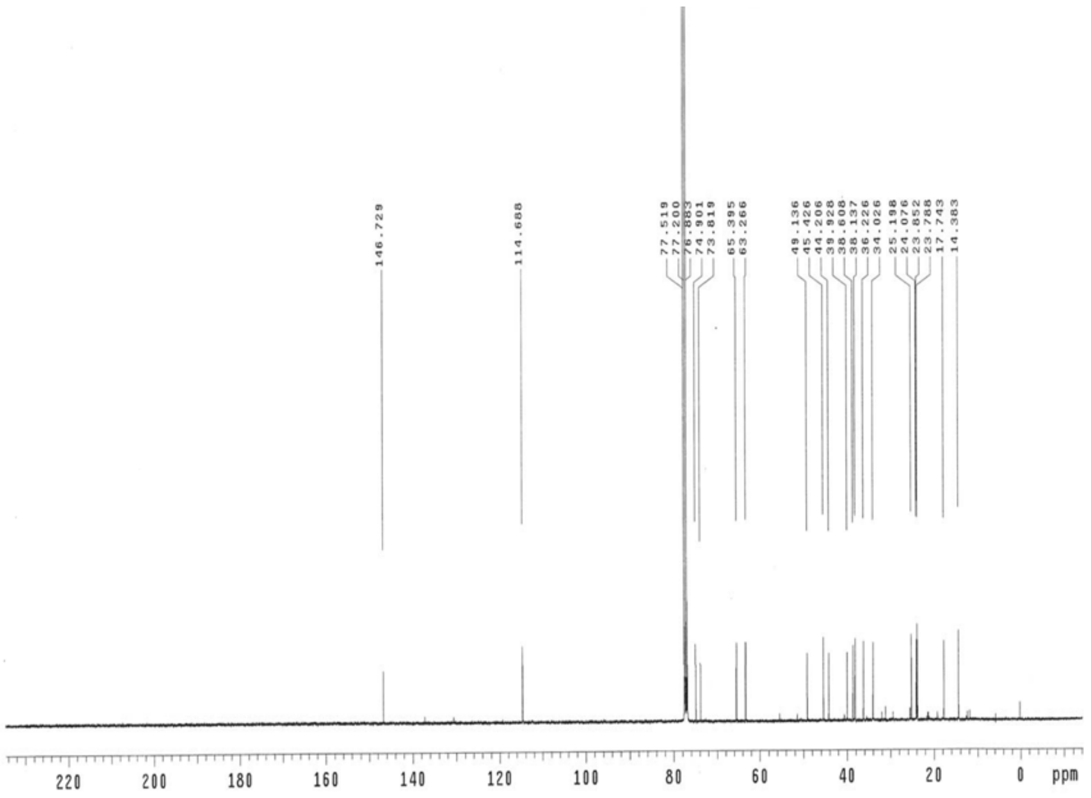

Figure S33. ^{13}C NMR spectrum of **5**.

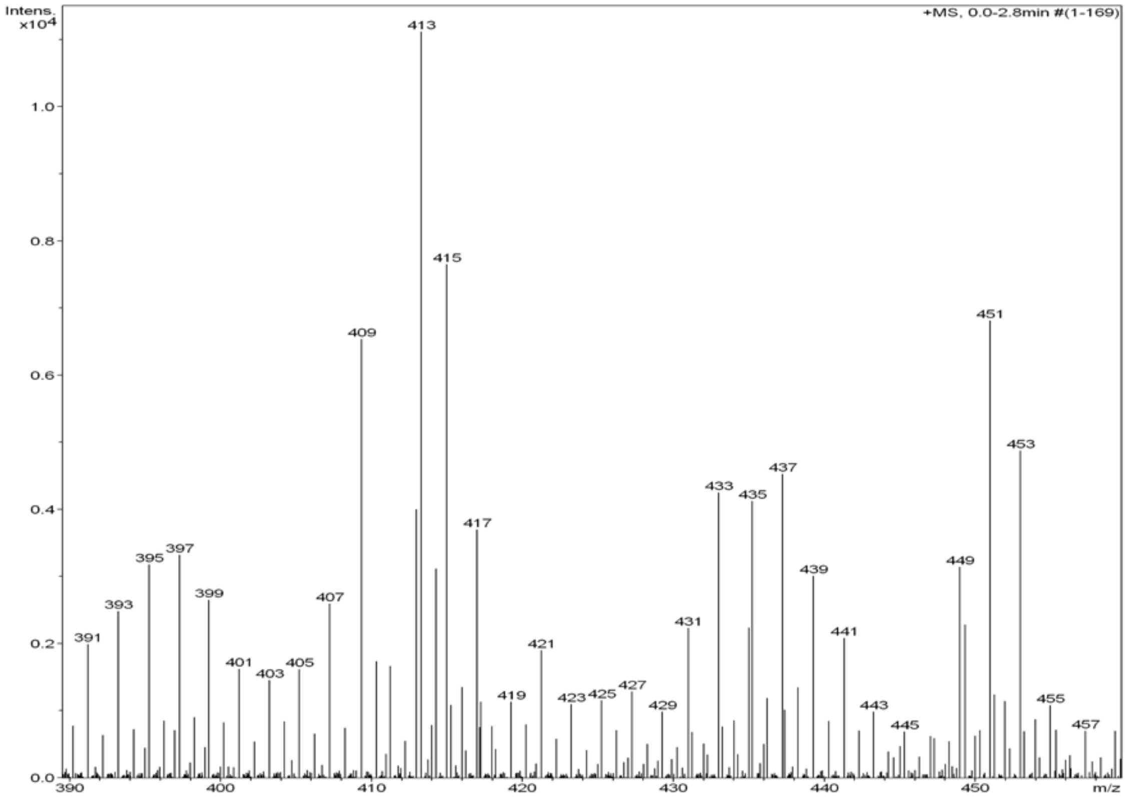

Figure S34. ESIMS spectrum of **6**.

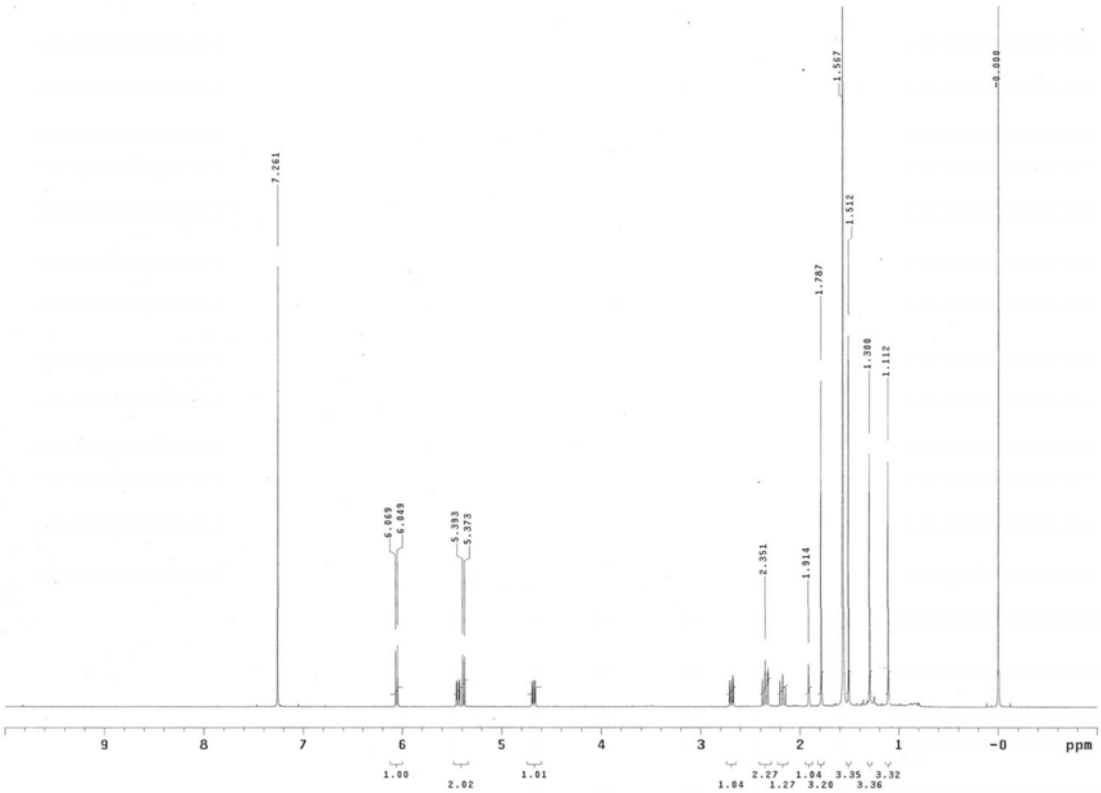

Figure S35. ^{1}H NMR spectrum of **6**.

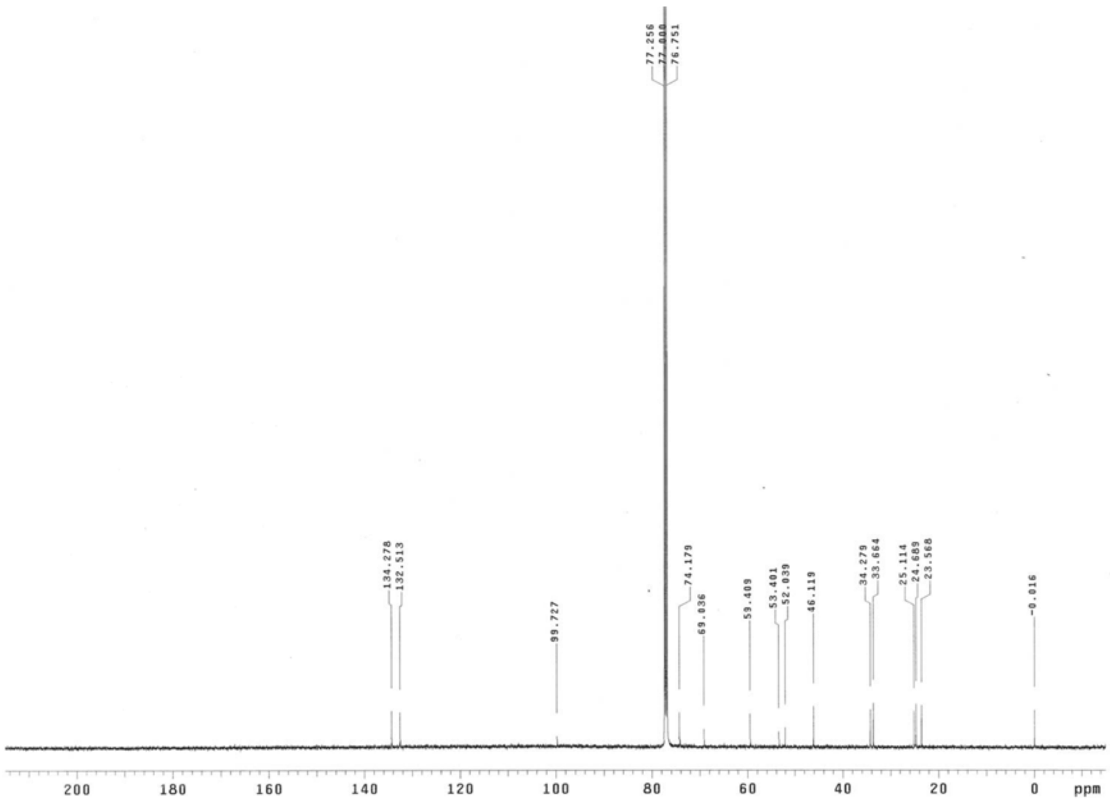

Figure S36.^{13}C NMR spectrum of **6**.

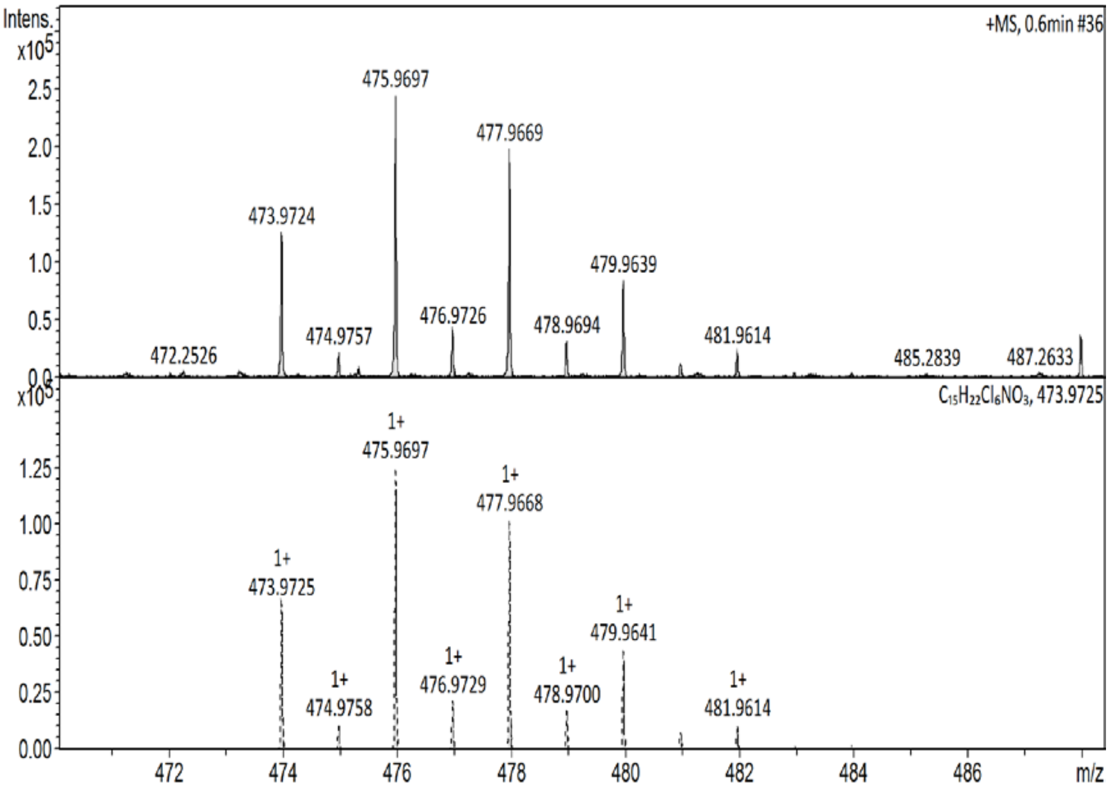

Figure S37. HRESIMS spectrum of **7**.

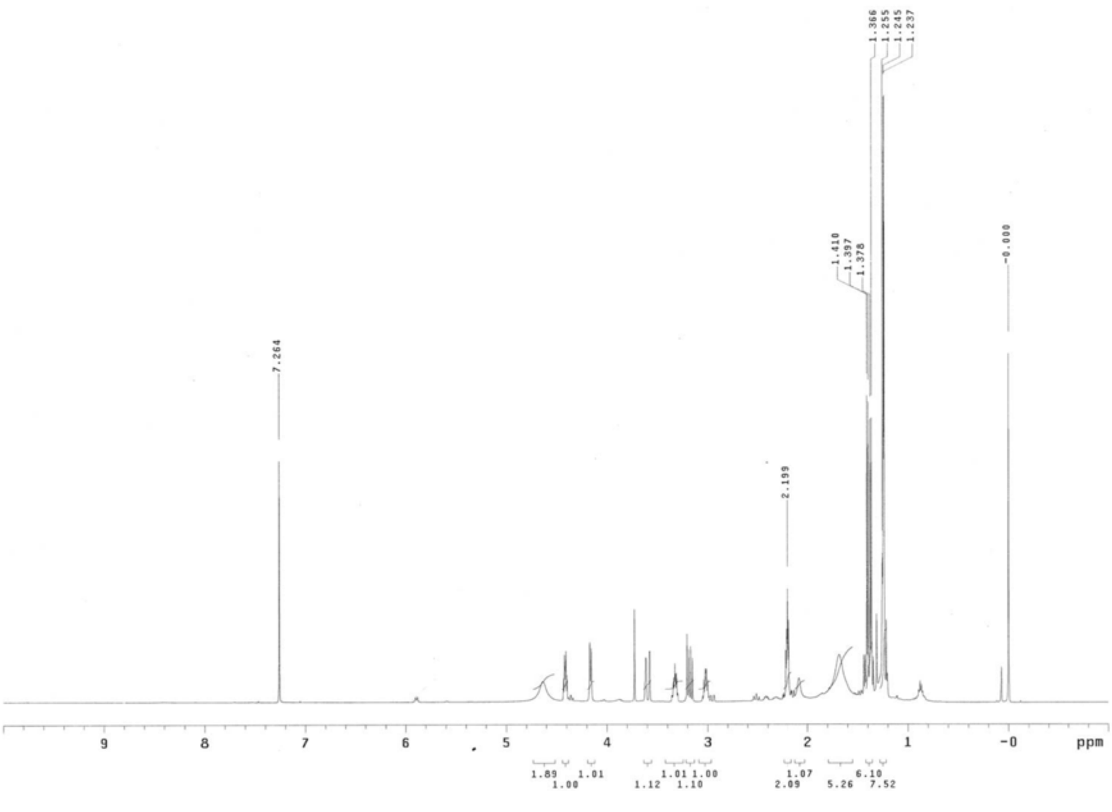

Figure S38. ^{1}H NMR spectrum of **7**.

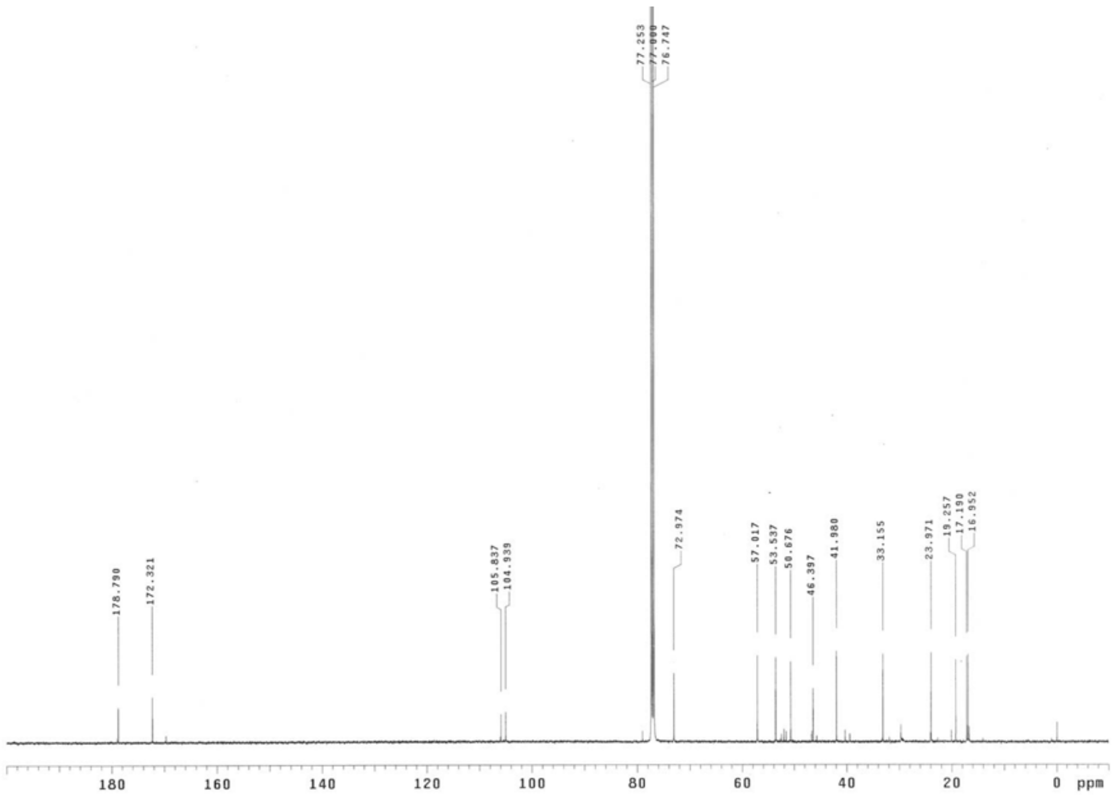

Figure S39. ^{13}C NMR spectrum of **7**.

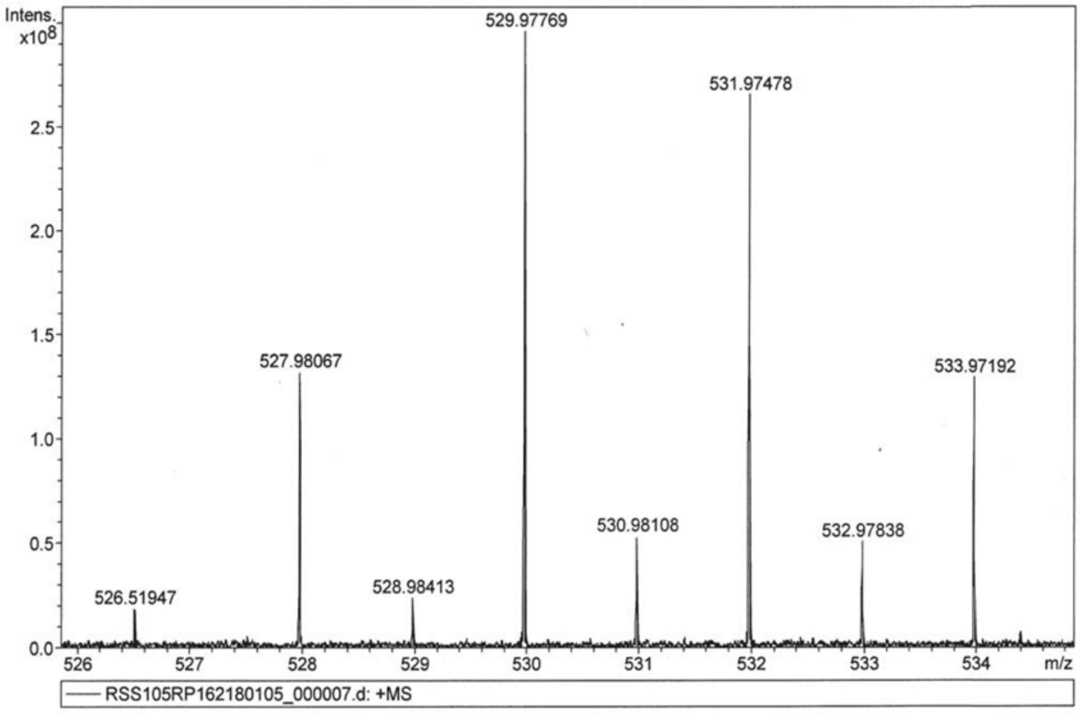

Meas. m/z	#	Formula	Score	m/z	err [mDa]	err [ppm]	mSigma	rdb	e⁻ Conf	N-Rule
527.98067	1	C 16 H 25 Cl 6 N Na O 4	100.00	527.98070	0.02	0.05	43.8	1.5	even	ok

Figure S40. HRESIMS spectrum of **8**.

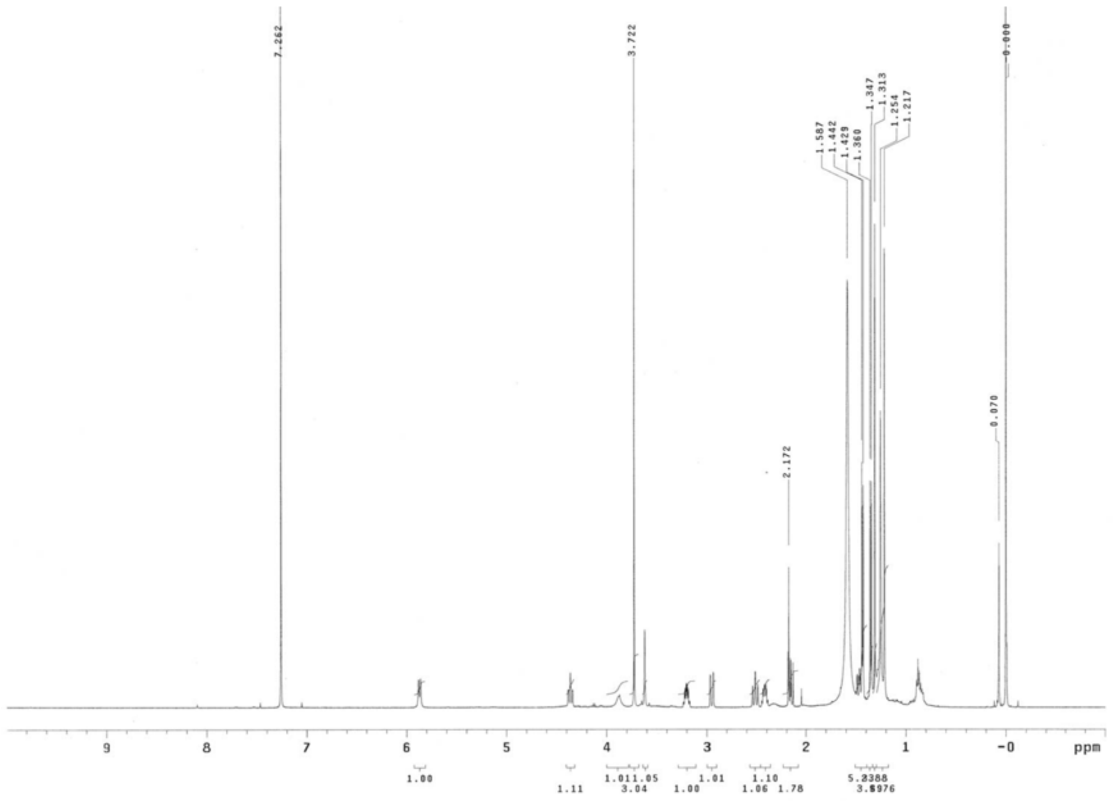

Figure S41. ^{1}H NMR spectrum of **8**.

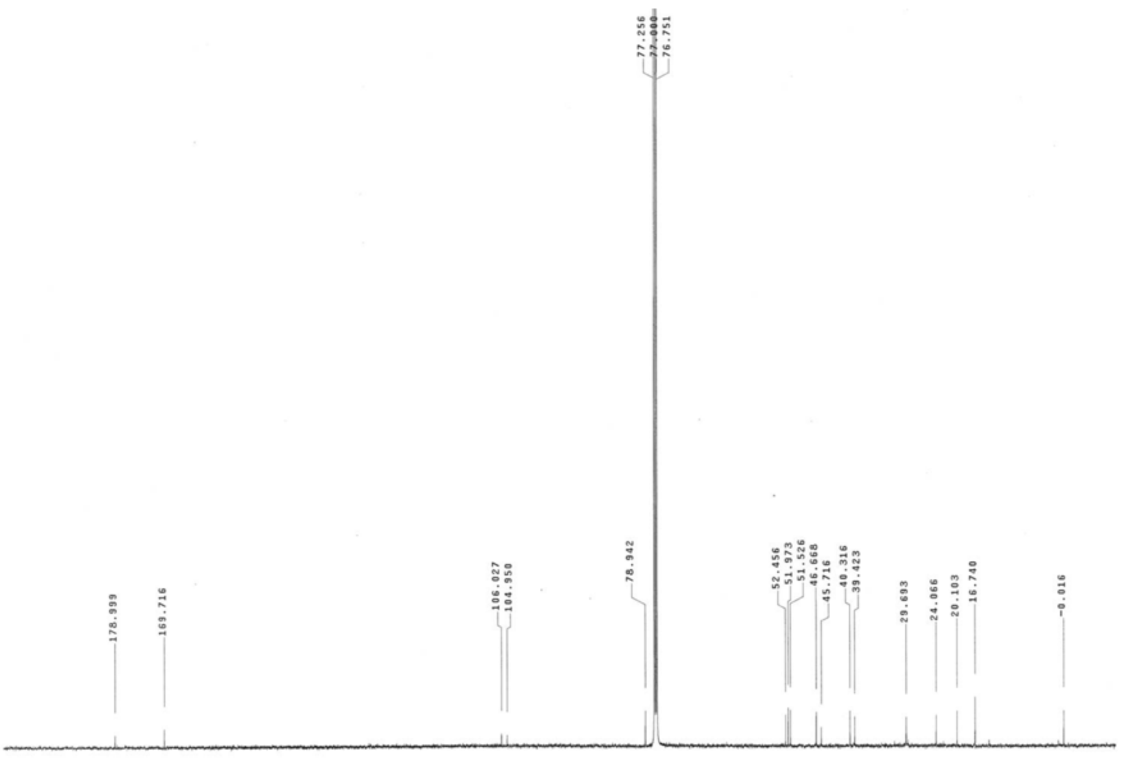

Figure S42. ^{13}C NMR spectrum of **8**.

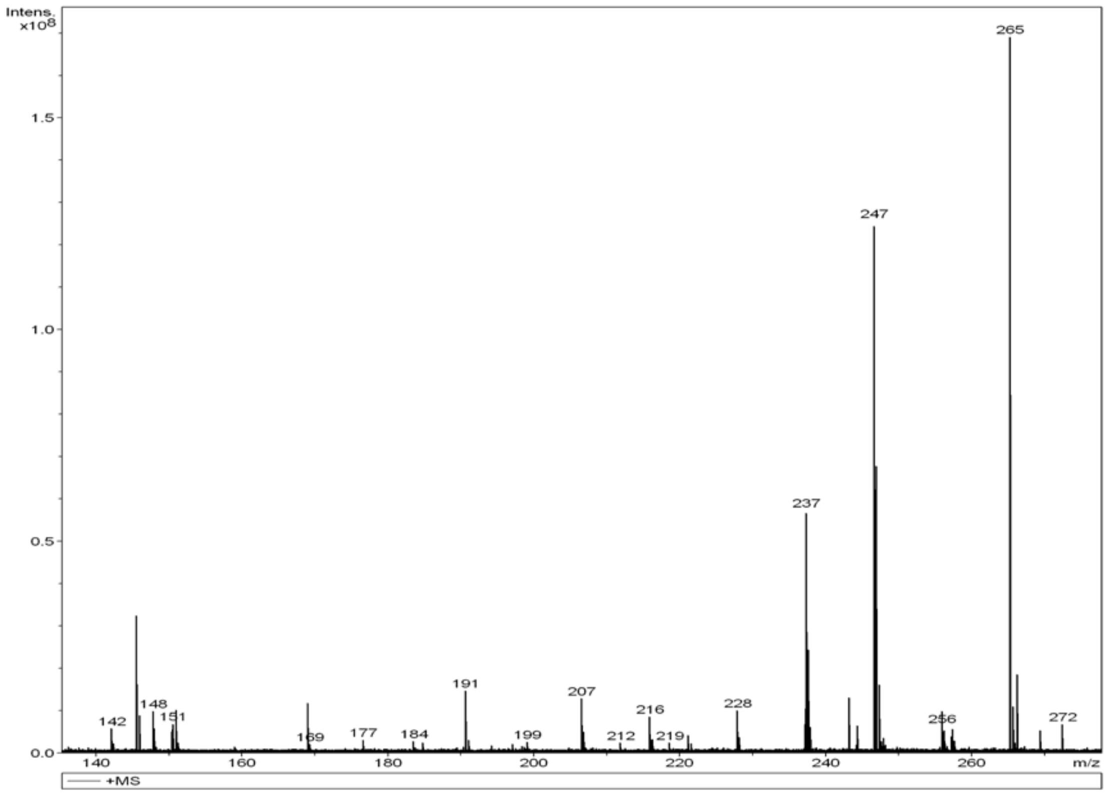

Figure S43. ESIMS spectrum of **9**.

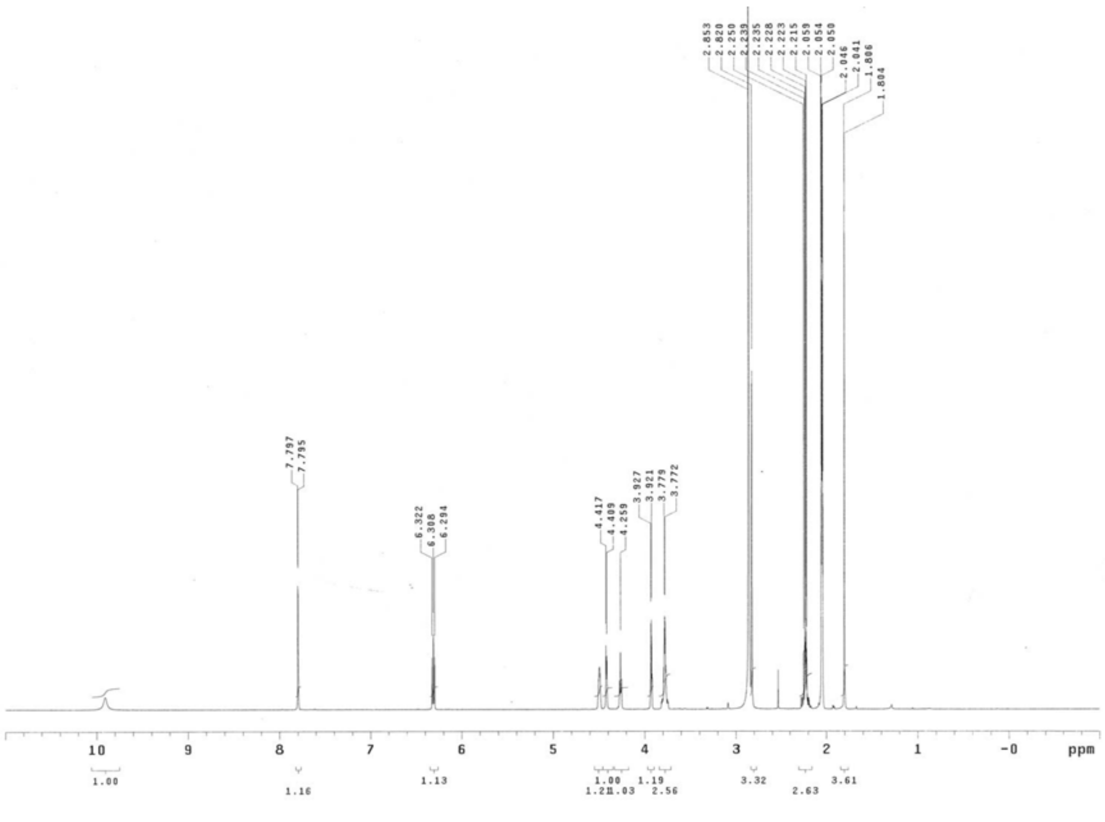

Figure S44. ^{1}H NMR spectrum of **9**.

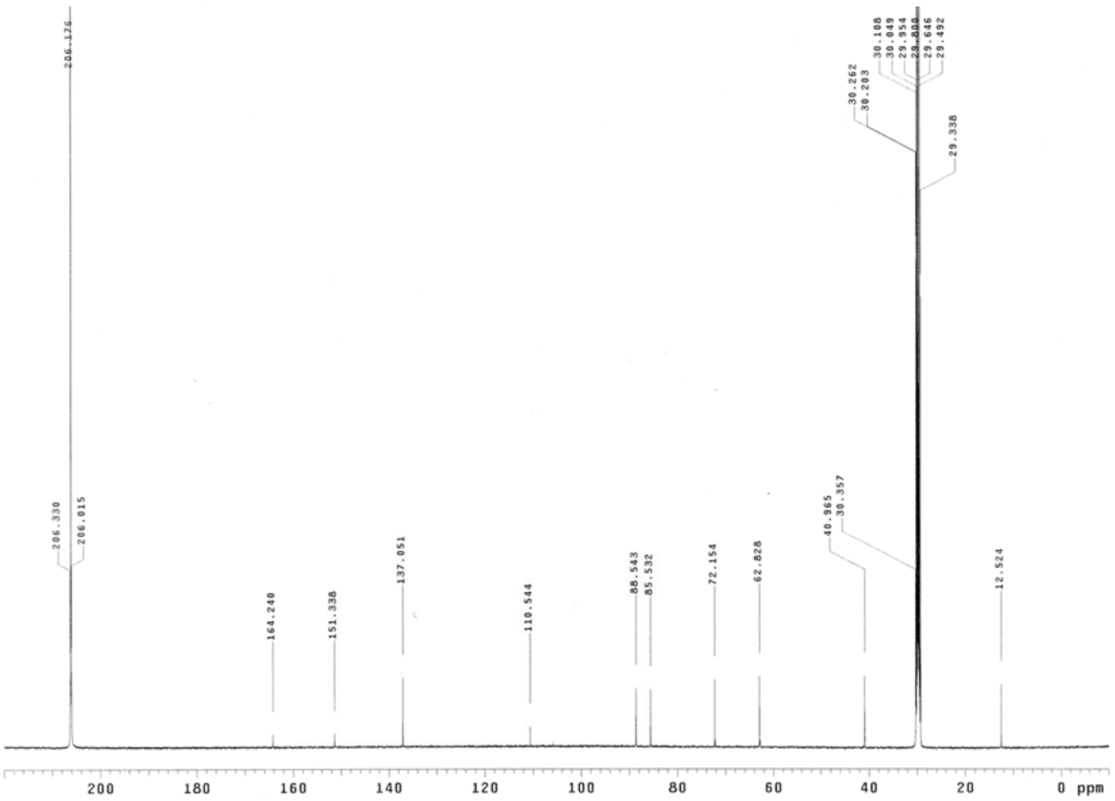

Figure S45. ^{13}C NMR spectrum of **9**.
